# Ultrasound‐Responsive HBD Peptide Hydrogel with Antibiofilm Capability for Fast Diabetic Wound Healing

**DOI:** 10.1002/advs.202406022

**Published:** 2024-09-09

**Authors:** Lanlan Zong, Runxin Teng, Huiqi Zhang, Wenshang Liu, Yu Feng, Zhengmao Lu, Yuxiao Zhou, Zhen Fan, Meng Li, Xiaohui Pu

**Affiliations:** ^1^ State Key Laboratory of Antiviral Drugs Henan Province Engineering Research Center of High Value Utilization to Natural Medical Resource in Yellow River Basin School of Pharmacy Henan University N. Jinming Ave. Kaifeng 475004 P. R. China; ^2^ Department of Polymeric Materials School of Materials Science and Engineering Tongji University 4800 Caoan Road Shanghai 201804 P. R. China; ^3^ Department of Dermatology Shanghai Children's Medical Center Shanghai Jiaotong University School of Medicine Shanghai 200127 P. R. China; ^4^ Department of Gastrointestinal Surgery The First Affiliated Hospital of Naval Medical University Shanghai 200433 P. R. China; ^5^ Department of Gynaecology and Obstetrics, Shanghai Key Laboratory of Anesthesiology and Brain Functional Modulation, Clinical Research Center for Anesthesiology and Perioperative Medicine, Translational Research Institute of Brain and Brain‐Like Intelligence Shanghai Fourth People's Hospital, School of Medicine Tongji University Shanghai 200434 P. R. China

**Keywords:** antibiofilm, deep delivery, diabetic wound, HBD peptide, ultrasound‐responsive hydrogel

## Abstract

Despite advancements in therapeutic agents for diabetic chronic wounds, challenges such as suboptimal bioavailability, intricate disease milieus, and inadequate delivery efficacy have impeded treatment outcomes. Here, ultrasound‐responsive hydrogel incorporated with heparin‐binding domain (HBD) peptide nanoparticles is developed to promote diabetic wound healing. HBD peptide, derived from von Willebrand Factor with angiogenic activity, are first engineered to self‐assemble into nanoparticles with enhanced biostability and bioavailability. Ultrasound responsive cargo release and hydrogel collapses are first verified through breakage of crosslinking. In addition, desired antioxidant and antibacterial activity of such hydrogel is observed. Moreover, the degradation of hydrogel under ultrasound stimulation into smaller fragments facilitated the deeper wound penetration of ≈400 µm depth. Complete wound closure is observed from diabetic mice with chronic wounds after being treated with the proposed hydrogel. In detail, in vivo studies revealed that hydrogels loaded with HBD peptide nanoparticles increased the levels of angiogenesis‐related growth factors (VEGF‐A, CD31, and α‐SMA) to effectively accelerate wound repair. Overall, this study demonstrates that ultrasound‐responsive HBD peptide hydrogel provides a synergistic therapeutic strategy for external biofilm elimination and internal effective delivery for diabetic wounds with biofilm infection.

## Graphical abstract

Herein, an ultrasound‐responsive hydrogel is constructed, designed for controlled drug release through the disruption of its network under ultrasound stimulation. Additionally, ultrasound facilitates the removal of wound biofilms and the deep delivery of encapsulated HBD peptide nanoparticles and antimicrobial agents, thereby completely eradicating bacteria and promoting angiogenesis, synergistically enhancing wound healing in diabetic infections.

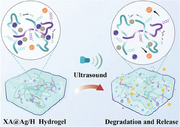

## 1 Introduction

Diabetes, a chronic metabolic disorder of escalating prevalence, stands as a leading cause of global morbidity and mortality, with forecasts estimating a staggering rise to 643 million and 783 million affected individuals by 2030 and 2045, respectively.^[^ ^1^ ^]^ Compellingly, chronic wounds are a frequent comorbidity in diabetes, exacerbating the risk of infection, complications, limb amputation, and even death.^[^ ^2^ ^]^ The wound healing cascade, typically orchestrated through hemostasis, inflammation, proliferation, and remodeling, is markedly disrupted in diabetic contexts. Factors such as heightened susceptibility to infections, biofilm formation, persistent inflammation, compromised vascular integrity, and impaired tissue remodeling significantly thwart the healing trajectory.^[^ ^3^ ^]^ During last decade, there have been a plenty of advancements in therapeutic agents for treating chronic wounds have been developed.^[^ ^4^ ^]^ However, diabetic wounds, particularly those with biofilm infections, present a formidable challenge due to microbial colonization and the development of multi‐drug resistance (MDR), which severely undermines the bioavailability and therapeutic potency of existing pharmaceuticals.^[^ ^5^ ^]^ To surmount these obstacles, there has been a surge in research focusing on the synergistic application of exogenous physical therapies, including photothermal therapy (PTT) and photodynamic therapy (PDT), in conjunction with bio‐patches, microneedles, and drug‐laden hydrogels.^[^ ^6^ ^]^ Yet, the efficacy of light‐based therapies is often curtailed by the limited penetration depth and the barrier posed by biofilms, necessitating alternative strategies for deep‐seated infections. Moreover, the potential for thermal damage to adjacent tissues with laser‐induced PTT necessitates caution.^[^ ^7^ ^]^

Compared to the use of light in the treatment, ultrasound with better penetration has emerged as a promising adjunct for drug delivery and tissue repair.^[^ ^8^ ^]^ In addition, ultrasound have been recognized for their ability to reduce interstitial pressure and decrease bacterial load through biofilm destruction.^[^ ^9^ ^]^ Due to the diverse physicochemical properties and versatile functionality, hydrogel has been widely applied in conjunction with ultrasound for biomedical applications. Among various building blocks of hydrogel, as a polysaccharide derived from the fermentation of glucose or sucrose by Xanthomonas campestris, Xanthan gum has been widely applied to form hydrogel as drug delivery systems, wound dressings, and scaffolds for tissue engineering.^[^ ^10^ ^]^ In addition, through combination with additional agents, ultrasound‐responsive xanthan gum hydrogel has also been developed to tailor the cargo release kinetics.^[^ ^11^ ^]^ Moreover, injectable Xanthan gum hydrogel was also synthesized and applied for wound healing to increase the accuracy of treatment.^[^ ^12^ ^]^ Therefore, it would be beneficial to endow Xanthan gum hydrogel with ultrasound responsiveness and injectability in conjunction with exogenous ultrasound to disrupt biofilms in diabetic wounds to enhance drug penetration.

Meanwhile, impaired angiogenesis in diabetic wounds also poses a significant challenge, hindering the wound healing process and further exacerbated by the presence of biofilms.^[^ ^13^ ^]^ Hyperglycemia induced deficiencies in vascularization not only impede the delivery of essential nutrients and oxygen to the wound site but also disrupt the continuous supply of growth factors, which are indispensable for effective wound healing. This vascular regeneration disruption exacerbates the challenges of wound closure in diabetic wounds.^[^ ^14^ ^]^ In the realm of wound healing and vascularization, the hemostatic protein von Willebrand factor (VWF) has been studied to be able to regulate angiogenesis.^[^ ^15^ ^]^ The HBD peptide derived from heparin binding domain of VWF protein with amino acid sequence of YIGLKDRKRPSELRRIASQVKYA has been identified as a key mediator, interacting with a spectrum of growth factors to influence angiogenesis.^[^ ^15^ ^]^ Meanwhile, such HBD peptide could be designed and modified with additional amino acids to become amphiphilic, which would self‐assemble into nanoparticles with enhanced biostability and bioavailability to promote angiogenesis.

**Scheme 1:**
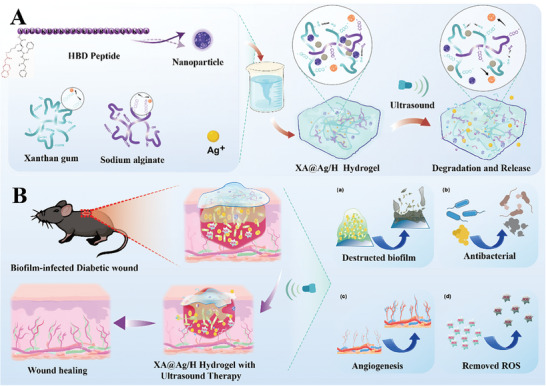
Ultrasound‐responsive HBD peptide Hydrogel for Fast Diabetic Wound Healing. A: Synthesis process of XA@Ag/H hydrogel and ultrasound induced cargo release and hydrogel degradation. B: The deep penetration of hydrogel for scavenging of biofilm and accelerated diabetic wound healing through enhanced antibacterial effects, angiogenesis, and removal of ROS.

In this work, we have developed a type of ultrasound‐responsive hydrogel incorporated with heparin‐binding domain (HBD) peptide nanoparticles for diabetic wound healing. At first, the N‐terminal of HBD peptide was further modified with hydrophobic Nap‐FF(K) as Nap‐FF(K)‐YIGLKDRKRPSELRRIASQVKYA to initiate self‐assembly into nanoparticles as shown in **Scheme** **1**. Concurrently, xanthan gum and sodium alginate are pre‐mixed with silver ions and HBD peptide nanoparticles for further cross‐linking. Afterwards, Ca^2+^ ions were applied to initiate the cross‐linking to form robust 3D dual‐network skeleton structure (XA@Ag/H hydrogel). Concurrently, ultrasound stimulation facilitates the release of degraded xanthan gum, which serves to actively neutralize reactive oxygen species (ROS), thereby alleviating oxidative stress within the diabetic wound environment. In tandem with this, the encapsulated silver ions and HBD peptide nanoparticles are concurrently liberated, endowing the system with potent anti‐infective capabilities and fostering angiogenesis. Moreover, the degradation of hydrogel under ultrasound stimulation into smaller fragments facilitated the deeper wound penetration of ≈400 µm depth. In vivo studies utilizing full‐thickness skin defect models in diabetic mice have demonstrated that the XA@Ag/H+US therapeutic strategy not only achieves profound drug penetration but also significantly accelerates the healing process of diabetic wounds with biofilm infection.

## 2 Results and Discussion

### 2.1 Synthesis and Characterization of XA@Ag/H Hydrogels

**Figure 1:**
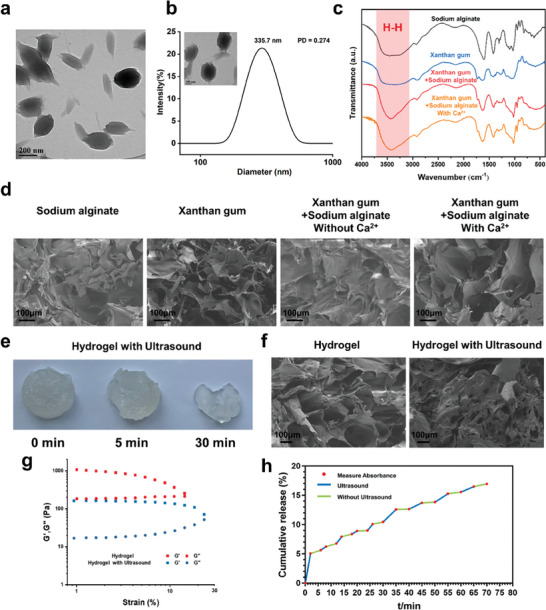
Preparation and characterization of HBD peptide and Ultrasound‐responsive hydrogel. a) Scanning electron microscopy (TEM)of HBD peptide nanoparticles. b) Dynamic light astigmatism (DLS)of HBD peptide nanoparticles. c) Infrared spectra of different hydrogels. d) TEM images of different hydrogels. e) Morphology changes of hydrogels after ultrasound. f) Changes of TEM images of hydrogels before and after ultrasound. g) Rheological curve of hydrogel. h) Release of ultrasonic responsive hydrogel.

Modified HBD peptides were first self‐assembled into prolate spheroid nanoparticles, as depicted in **Figure** **1**a. The hydrodynamic diameter of these nanoparticles, as determined by dynamic light scattering, was ≈335.7 nm (Figure 1b), confirming the peptide's self‐assembly properties. As shown in Figure S1 (Supporting Information), the stability of HBD peptide nanoparticles was also evaluated using dynamic light scattering. Within PBS buffer solution (pH 7.4), no obvious change was observed of the derived count rate of HBD peptide nanoparticles, indicating desired stability of self‐assembled peptide nanoparticles. Meanwhile, under external ultrasound stimulation and acidic aqueous solution to mimic infected wound, the derived count rate of HBD peptide nanoparticles was obviously reduced. With the responsive disassembly of HBD peptide nanoparticles at wound site, more HBD peptide monomers were released to restore its original biological activity. Therefore, the self‐assembled HBD peptide nanoparticles could enhance the peptide stability and maintain its original bioactivity through responsive release at wound site, which could increase the bioavailability of HBD peptide and promote wound healing. Fourier‐transform infrared spectroscopy (FTIR) analysis revealed a significant shift in the characteristic peak for the carboxylate (‐COO^−^) groups within the xanthan gum/sodium alginate composite. Specifically, the peak shifted from 1635 to 1630 cm^−1^ upon the addition of Ca^2+^ ions, as shown in Figure 1c, indicating the coordination interactions between the ‐COO^−^ groups and Ca^2+^ ions. Moreover, the peak associated with the stretching vibration of hydroxyl (O‐H) groups in the range of 3700–3000 cm^−1^ was observed to be broader and more intense in the XA hydrogel compared to control groups. This suggests that the presence of Ca^2+^ ions significantly enhances hydrogen bonding interactions between water molecules and the xanthan gum/sodium alginate matrix, contributing to the hydrogel's structural integrity.^[^ ^16^ ^]^ Notably, the unique 3D hollow structure of the hydrogel, as illustrated in Figure 1d, was exclusively observed in the xanthan gum/sodium alginate composite crosslinked with Ca^2+^ and subjected to freeze‐drying. This finding highlights the essential role of Ca^2+^ ions in the hydrogel's 3D architecture, which is crucial for its intended biomedical applications.

Moreover, injectable hydrogels can be more convenient to use via syringe for wound healing, allowing for precise treatment to irregular wound sites and enhanced therapeutic outcomes. To precisely regulate the gelation process, two syringes connected with a three‐way stopcock was applied, which could be utilized to adjust the physicochemical properties of formed hydrogel. Through optimization, injectable Xanthan GUM hydrogel was prepared as shown in Figure S2 (Supporting Information). Collectively, these results elucidate the molecular interactions and structural features of the Ca^2+^ crosslinked xanthan gum/sodium alginate hydrogel, providing a foundation for its further optimization and biomedical application.

### 2.2 Evaluating the Ultrasound Responsiveness of Hydrogel

In our study, we examined the effects of ultrasound‐induced hydrogel disruption at both macroscopic and microscopic levels. A progressive release of Ca^2+^ ions from the hydrogel was observed with increasing durations of ultrasound exposure, concomitant with a corresponding reduction in hydrogel mass (Figure S3, Supporting Information). Macroscopic observations indicated that hydrogel degradation intensified with prolonged ultrasound application (Figure 1e). Scanning electron microscopy (SEM) analysis revealed that the hydrogel's original homogeneous and porous structure was significantly altered upon ultrasound treatment, with the pores appearing to collapse and leading to fragmentation (Figure 1f). As shown in Figure S4 (Supporting Information), the XPS spectrum revealed that the amount of Ca2p was reduced by 20% after 5 min of ultrasound stimulation, demonstrating that the Ca^2+^ induced cross‐linking could be disrupted by external sonication. Additionally, rheological analysis was conducted to evaluate changes in the viscoelastic properties of the hydrogels before and after ultrasound treatment (Figure 1g). Throughout the tested frequency range, the storage modulus (G') consistently exceeded the loss modulus (G''), a characteristic indicative of a stable gel. The hydrogel exhibited a higher modulus before ultrasound stimulation, suggesting a structural weakening of the hydrogel network post‐treatment.

Furthermore, ultrasound facilitated the controlled release of encapsulated cargo from the hydrogel, as shown in Figure 1h. After the cessation of the ultrasound stimulus, cargo release continued at a sustained but reduced rate. Compared to the inherent release profile of the hydrogel, which released only 35.23% of the cargo over a week (Figure S5, Supporting Information), this rate may be insufficient to provide the necessary therapeutic levels of bioactive agents for effective wound healing. In the current work, under intermittent ultrasound stimulation, we achieved a cumulative release of ≈16.94% within 70 min, which could significantly enhance the therapeutic efficacy of bioactive hydrogels for accelerated wound healing. Ultrasound‐responsive hydrogel systems offer the unique capability to modulate the release profile of therapeutic agents in a precise manner by fine‐tuning the parameters of ultrasound application. This customizable feature is particularly advantageous for providing tailored treatment strategies that cater to the individual needs of patients with diabetic chronic wounds.

### 2.3 Biocompatibility, Hemocompatibility, Proliferation, and Migration In Vitro

**Figure 2:**
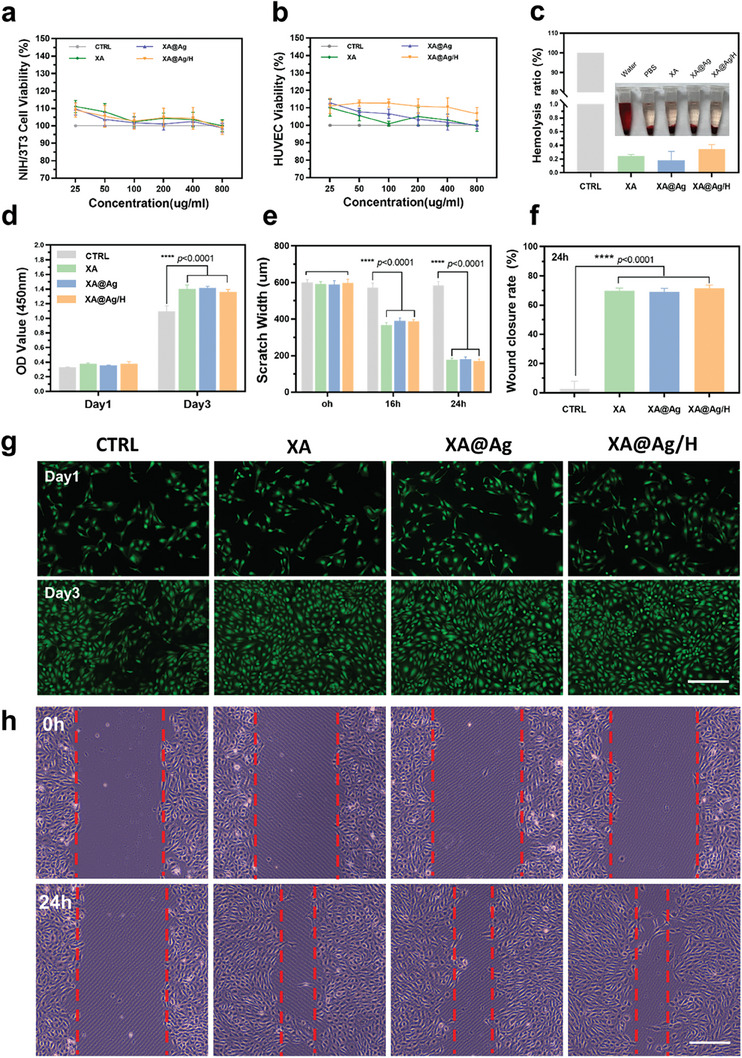
In vitro Biocompatibility and migration assessment of hydrogels. a) NIH/3T3 cells and b) HUVECs viability of hydrogels treated for 24 h. c) Hemolytic ratios and photographs of hemolytic activity assay of hydrogels, PBS as a negative control, and water as a positive control. d) Proliferation of NIH/3T3 treated with XA, XA@Ag, and XA@Ag/H for 1and 3 days (n = 3). e) and f) corresponding scratch widths and closure rate. g) Live/Dead staining of HUVECs after culture with hydrogels for 1 and 3 days. Scale bar = 300µm. h) Scratch healing of HUVECs. Scale bar = 300µm. Data represent mean ± SD; **** *p *< 0.0001.

To assess the biocompatibility of such hydrogel, a Live/Dead assay was conducted on NIH/3T3 cells in contact with various hydrogels, revealing that cell viability was maintained comparably after 48 h of culture with XA, XA@Ag, and XA@Ag/H hydrogels (Figure S6, Supporting Information). This was further substantiated by the CCK‐8 assay, which quantitatively evaluated the cytotoxicity of NIH/3T3 and HUVEC cells cultured with or without the hydrogels. The results indicated a survival rate exceeding 100% for cells exposed to different concentrations of hydrogel, with no significant loss in viability even at a high concentration of 800 µg mL^−1^, suggesting the composite hydrogel is non‐toxic and promotes cell proliferation, which is conducive to wound healing (**Figure** **2**a‐b). Additionally, the hemolysis assay was performed to gauge the hemocompatibility of the hydrogel, with the hemolytic rate (HR) being a critical parameter for biomaterial evaluation. The HR for all hydrogels was found to be less than 1.0%, indicating excellent blood compatibility (Figure 2c).

To further investigate the hydrogel's role in cell proliferation, the CCK‐8 assay was used to measure the viability of NIH/3T3 cells cultured with or without hydrogel (50 µg mL^−1^). Following a 3‐day incubation, the hydrogel‐covered cells showed significantly higher viability than the control group (Figure 2d). According to live/dead staining of HUVECs, the density of the cells after three days of co‐culture with hydrogels was greater than that without hydrogels in the control group (Figure 2g). This aligns with the previously discussed toxicity results, confirming the composite hydrogel's significant capacity to enhance the proliferation of both NIH/3T3 and HUVEC cells, a key factor in the wound healing process. The impact of the hydrogel on cell migration was evaluated by exposing HUVECs to hydrogel dilutions and observing their migration using a fluorescence microscope. HUVECs treated with the hydrogels displayed faster gap closure rates compared to those treated with PBS alone (Figure 2h). After 24 h of co‐culture, the scratch width was reduced to ≈200 µm, and the healing rate achieved 70.8% (Figure 2e‐f). These results highlight the XA@Ag/H hydrogel's exceptional cytocompatibility, along with its ability to enhance cell proliferation and migration, thereby fostering a more favorable environment for cellular growth and wound repair. Collectively, these findings underscore the XA@Ag/H hydrogel's excellent biocompatibility at the cellular level, its significant augmentation of cell proliferation and migration, and its potential to provide an optimal environment for tissue regeneration and wound healing.

### 2.4 Antioxidant Efficacy and Blood Cutting Studies

**Figure 3:**
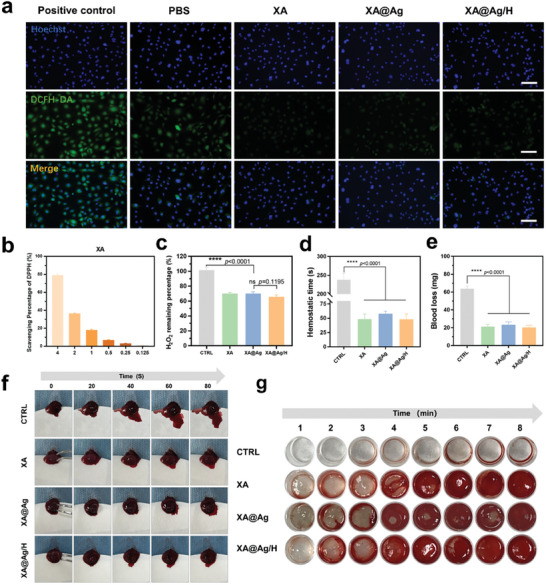
Antioxidant activity and hemostatic effect of hydrogels. a) ROS fluorescence images of NIH‐3T3 cells after different treatments. Scale bar = 200µm. b) Scavenging percentage of DPPH radicals at different concentrations of Xanthan gum. c) Scavenging effect of different hydrogels with 1mg mL^−1^ concentration on hydrogen peroxide (n = 4). d) Hemostasis time of liver wounds after different treatments (n = 3). e) Total bleeding volume from the damaged hepatic tissues (n = 3). f) Representative images of liver tissues exhibiting hemorrhaging administered with different hydrogels at time points of 0,20,40,60, and 80s. g) Images of evolution of thrombus formation in relation to time, under the influence of assorted hydrogel interventions. Data represent mean ± SD; ns *p* > 0.1, ****p < 0.0001.

Reactive oxygen species (ROS) are known to play both a beneficial and a detrimental role in diabetic wound healing, acting as both an asset and a liability, which reflects their complex and paradoxical involvement in the healing process.^[^ ^17^ ^]^ Moderate levels of ROS are essential for their bactericidal effects against pathogenic bacteria, thus positively contributing to wound healing. However, an overabundance of ROS can lead to increased oxidative stress, which can be harmful to the wound microenvironment and hinder the healing process. This fine balance between the beneficial and detrimental effects of ROS underscores the intricacies of their role in the management of diabetic wounds.^[^ ^18^ ^]^ Therefore, the timely neutralization of excess ROS is essential to mitigate unwarranted inflammation, promote a secondary anti‐inflammatory response, and facilitate the wound healing cascade. To assess the antioxidant capacity of the XA hydrogel, a free radical scavenging assay was conducted using the 2,2‐diphenyl‐1‐picrylhydrazyl (DPPH) radical as a standard. The XA hydrogel demonstrated a significant inhibitory effect on DPPH radicals at a concentration of 250 µg mL^−1^, with an increase in scavenging efficiency as the hydrogel concentration was progressively increased, as shown in **Figure** **3**b. This suggests a concentration‐dependent relationship between the DPPH scavenging ability of the XA hydrogel and its concentration. Moreover, the hydrogels showed a 60% scavenging rate for hydrogen peroxide at a concentration of 1 mg mL^−1^, as depicted in Figure 3c.

Anticoagulant therapy is a standard treatment for diabetic patients at risk for vascular complications such as cardiovascular disease and atherosclerosis.^[^ ^19^ ^]^ Therefore, even minor post‐debridement bleeding must be carefully considered.^[^ ^20^ ^]^ To evaluate the hemostatic properties of the hydrogel, blood samples were treated with the hydrogel and their clotting times were measured. Whole blood treated with the hydrogel showed no significant coagulation after 8 min, whereas a stable clot was formed within ≈4 min upon contact with the XA hydrogel. Notably, the XA@Ag hydrogel exhibited superior blood clotting efficacy, achieving clot formation in ≈2 min (Figure 3g), with no statistically significant difference compared to the XA@Ag/H group. Additionally, a mouse hepatic hemorrhage model was utilized to assess the hemostatic capability of the XA@Ag/H hydrogel. There was a marked reduction in blood loss in the XA@Ag/H hydrogel treated groups (20.33±2.67 mg) compared to the blank control (63.66±2.94 mg), with the XA hydrogel and XA@Ag group also demonstrating comparable hemostatic efficacy (21.33±2.33 and 23.33±3.67 mg, respectively) (Figure 3d‐e). Calcium (Ca) ions are recognized for their role in enhancing and hastening the process of blood clotting. They achieve this by aiding the synthesis of the prothrombinase complex, a crucial component that transforms prothrombin into thrombin, and then catalyzes the conversion of fibrinogen into fibrin.^[^ ^21^ ^]^ Therefore, it is plausible that the procoagulant effect observed may be attributed to the Ca ions within the hydrogel, which activate thrombin and initiate the coagulation cascade, thus providing an effective hemostatic response.

### 2.5 Evaluation of Antibacterial Activity and Antibiofilm Ability In Vitro

**Figure 4:**
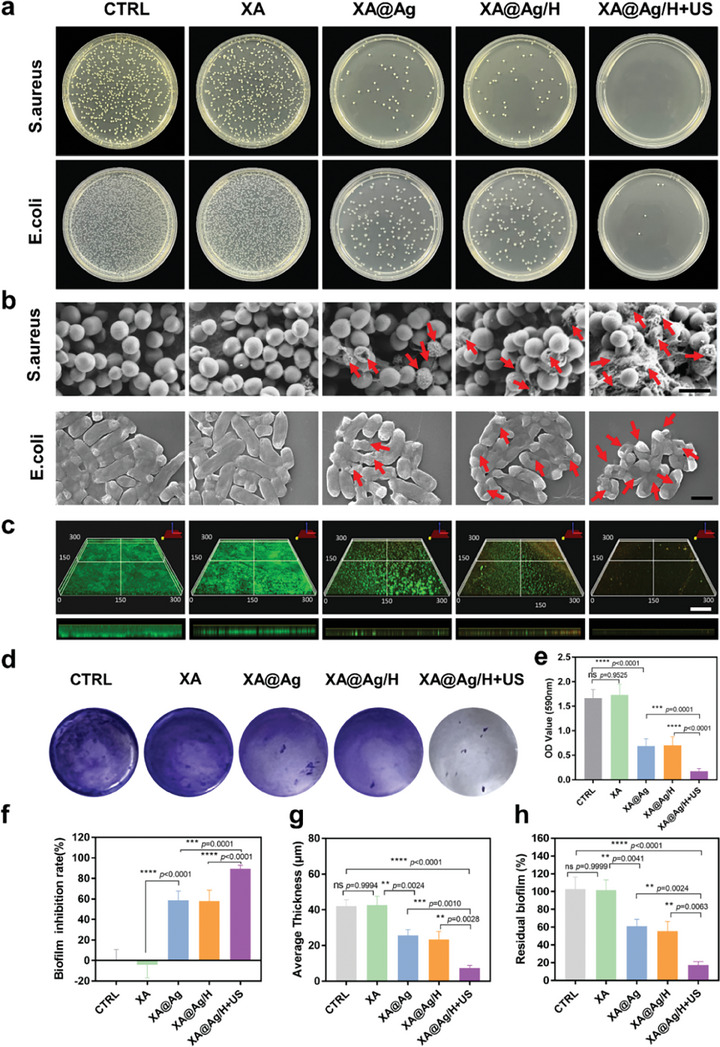
Evaluation of antibacterial and anti‐biofilm activities. a) Representative optical images of Staphylococcus aureus and Escherichia coli after different treatments. b) The pertinent scanning electron microscopy (SEM) visuals of Staphylococcus aureus and Escherichia coli organisms that underwent a spectrum of therapeutic interventions. Scale bar = 1µm. c) Representative images of stained Staphylococcus aureus biofilm scanned using confocal laser scanning microscopy (CLSM). Scale bar = 50µm. d) Representative photographs of crystal violet staining of S. aureus biofilms after different hydrogel treatments. e) Absorbance of remaining crystal violet dissolved in alcohol solution at 590nm. f) Inhibition rate of S. aureus biofilm after treatment with different hydrogels. g) and h) Corresponding thickness of biofilm and residual biofilm of each group. Data represent mean ± SD; ns *p* > 0.1, **p < 0.01, ***p < 0.001, ****p < 0.0001.

Considering the established antibacterial effects of ultrasound and its ability to facilitate the controlled release of silver ions, we subsequently investigated the in vitro antibacterial properties of the ultrasound‐responsive hydrogel. As depicted in **Figure** **4**a, the XA group and the control group exhibited nearly identical colony counts on agar plates, suggesting that XA alone has negligible resistance against E. coli and S. aureus. Conversely, hydrogels releasing silver ions demonstrated significant antibacterial activity, with the XA@Ag/H+US group outperforming others by eradicating 97.38% of S. aureus and 92.5% of E. coli (Figure S7, Supporting Information). Furthermore, incubation of the hydrogel with both bacteria for 48 h revealed, through growth curves, that the ultrasound‐responsive hydrogel group had the slowest rate of bacterial proliferation, suggesting an enhanced antibacterial effect attributed to ultrasound (Figure S8, Supporting Information). The observed antibacterial activity is likely due to the interaction between positively charged silver ions and the sulfhydryl (‐SH) groups on bacterial proteases, neutralizing their activity and leading to bacterial death.^[^ ^22^ ^]^ Additionally, the synergistic effect of ultrasound includes accelerating the release of silver ions and generating microbubbles that, upon implosive collapse, exert shear forces capable of damaging bacterial cell walls, thereby enhancing the sterilization effect. The XA@Ag/H+US hydrogel's superior antibacterial properties suggest its potential utility in treating bacterially infected wounds.

To elucidate the mechanism of bacterial destruction by the hydrogel, scanning electron microscopy (SEM) was utilized to scrutinize the morphological alterations in Staphylococcus aureus and Escherichia coli following treatment. (Figure 4b). Bacteria in the control and XA groups appeared smooth and intact, whereas those in the XA@Ag and XA@Ag/H groups showed surface irregularities, indicating that silver ions released without ultrasound have limited bactericidal effects. In stark contrast, the XA@Ag/H+US group exhibited pronounced membrane damage and extrusion of cellular contents, confirming that ultrasound treatment significantly enhances the antibacterial potency of hydrogel. Besides, to study the drug resistance after treated with silver ions in the form of hydrogel, continuous treatment of S. aureus with ciprofloxacin, silver ions solution, and silver ions loaded hydrogels was conducted. Obvious resistance was observed from S. aureus treated with ciprofloxacin for 5 consecutive days. Whereas, no obvious resistance was observed from S. aureus treated with silver ion solution and silver ion loaded hydrogel, demonstrating that silver ions in the form of aqueous solution and hydrogel are not prone to drug resistance (Figure S9, Supporting Information).

Biofilms are known to impede the penetration of antibiotics and other therapeutics into wounds, thereby thwarting their efficacy and contributing to the chronicity of diabetic wounds.^[^ ^12^, ^23^ ^]^ Given S. aureus's propensity for rapid transmission and biofilm formation, it was selected as a representative strain for anti‐biofilm assays. Crystal violet staining revealed that XA had no effect on established biofilms, whereas XA@Ag and XA@Ag/H treated biofilms exhibited reduced staining intensity, indicative of some biofilm clearance capacity (Figure 4d). Notably, the XA@Ag/H+US group displayed nearly complete biofilm eradication. Subsequent quantification using 95% ethanol to dissolve residual dye demonstrated an absorbance of 0.17 for the ultrasound group, compared to the control group's absorbance of 1.66 (Figure 4e). The calculated biofilm clearance rates were 55.39% and 55.14% for XA@Ag and XA@Ag/H, respectively, whereas the XA@Ag/H+US group achieved a remarkable 90.42% clearance rate (Figure 4f). 3D confocal laser scanning microscopy confirmed these findings, showing that hydrogels untreated by ultrasound could only partially disrupt the biofilm surface, leaving deeper bacteria viable and adherent (Figure 4c). Analysis of biofilm thickness and biomass revealed that the average biofilm thickness in untreated controls was 42 µm. In contrast, biofilms treated with the ultrasound‐responsive hydrogel exhibited a significantly reduced thickness of merely 7.3 µm, corresponding to an impressive clearance rate of 82.6%. These findings underscore the efficacy of ultrasound‐treated hydrogels in the elimination of biofilms, highlighting their potential utility in combating chronic infections, particularly those involving biofilm formation.

### 2.6 Assessment of Promoting Diabetic Wound Healing

**Figure 5:**
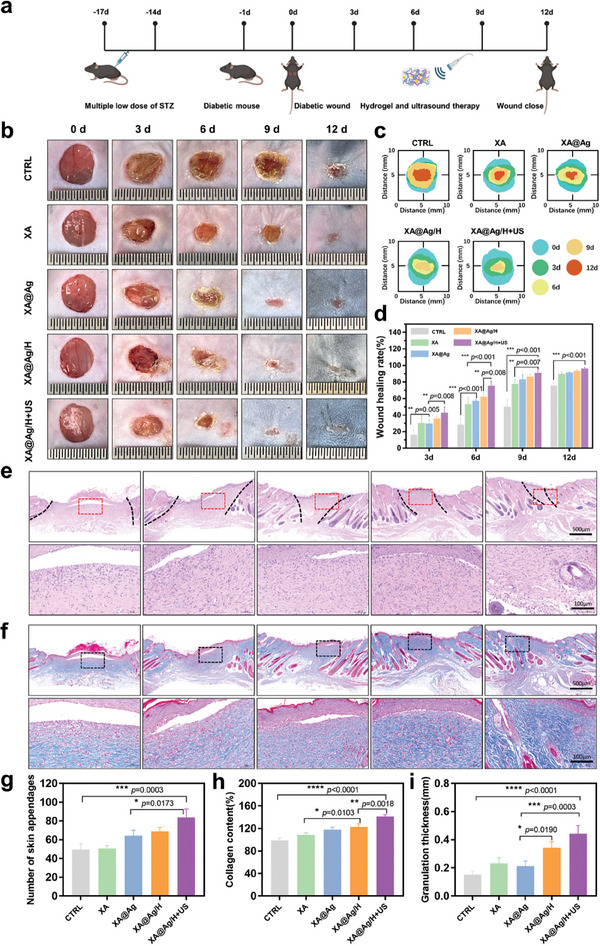
XA@Ag/H+US hydrogel promotes diabetic wound healing. a) Schematic diagram of the experimental protocol. b) Typical photographs illustrating the progression of the wound healing trajectory in a diabetic murine model. c) Traces of wound closure during 12 days. d) The rate of wound closure was determined by comparing the initial and subsequent digital photographs of the wounds. e) Hematoxylin and eosin‐stained (H&E) mice skin tissue of the wound area. f) Masson's trichrome staining of skin tissue in mouse wound area. g) Quantification of skin appendages (hair, sebaceous glands, and blood vessels) in the wound area on day 12. h) Quantification of Granulation tissue thickness in different groups on day 6. i) Quantification of collagen deposition density in different groups on day 12 (n = 3). Data represent mean ± SD; *p < 0.05, **p < 0.01, ***p < 0.001, ****p < 0.0001.

To substantiate the in vivo wound healing efficacy of the XA@Ag/H+US hydrogel, a diabetic wound model featuring a full‐thickness skin defect was established (**Figure** **5**a). Throughout the model establishment, we routinely monitored the blood glucose levels in the mice to confirm the stability of the induced diabetic condition (Figure S10, Supporting Information). Analysis of the digital wound photographs (Figure 5b) and the accompanying healing simulation diagram (Figure 5c) revealed that the XA@Ag/H+US group showed a markedly smaller wound area compared to other groups by day 6 post‐treatment. Quantitative assessment depicted in Figure 5d indicated that the average wound closure rates on day 6 were 62.45% for the XA@Ag/H group and 75.53% for the XA@Ag/H+US group, suggesting a synergistic enhancement of wound healing with the combined application of hydrogel and ultrasound. This accelerated healing is likely due to the ultrasound‐induced degradation of the hydrogel, which promotes the deep penetration of the hydrogel along with its therapeutic payload—silver ions and HBD peptide nanoparticles—into the skin tissue, thus improving the hydrogel's bioavailability. Moreover, by day 12, the average wound healing area for the XA@Ag/H+US group had significantly increased to ≈96.2%, indicating the most rapid healing rate and a pronounced acceleration in wound closure compared to the control group, which achieved 75.83% healing.

Re‐epithelialization constitutes a pivotal and initial phase in the cascade of skin wound healing, antecedent to dermal restoration.^[^ ^24^ ^]^ This process is paramount in averting excessive transepidermal water efflux and preempting wound sepsis by affording a provisional functional barrier to the afflicted tissue.^[^ ^25^ ^]^ To validate the regeneration of the epithelium and the concomitant formation of granulation tissue, hematoxylin, and eosin (HE) staining and Masson's trichrome staining were systematically applied to the wound specimens across all experimental cohorts on days 6 and 12 post‐wounding. On the sixth day of the analysis, a discernible discrepancy in the dimensions of the wound bed was observed upon histological examination, with the control group exhibiting the most pronounced wound aperture, whereas the wounds in the treatment groups demonstrated a significant reduction in size (Figure S11, Supporting Information). Elaborating further, Figure 5e delineates that the XA@Ag/H+US group's wound tissue sections displayed a relatively intact epithelial layer and an augmented count of dermal appendages, encompassing sebaceous glands, hair follicles, and neovascular structures (Figure 5g). In stark contrast, the tissue sections from wounds in the other experimental groups presented with an incomplete epithelium. Notably, wounds subjected to PBS treatment persisted in a state of arrested healing after 12 days, marked by expansive wound gaps and a deficient epithelial layer.

Granulation tissue is integral to the reparative process of wounds characterized by inflammation, serving as a bed for re‐epithelialization and a scaffold for neovascularization.^[^ ^26^ ^]^ Given its significance, the assessment of granulation tissue thickness is a critical parameter for gauging the efficacy of wound repair. As depicted in Figure 4i, the XA@Ag/H+US treatment cohort exhibited a pronounced increase in granulation tissue thickness by the sixth day of the study, surpassing that of the XA@Ag/H, XA@Ag, and XA groups, which suggests a superior reparative effect conferred by the XA@Ag/H+US treatment modality. Furthermore, the deposition of collagen, a cardinal component of the extracellular matrix, is positively correlated with the wound healing trajectory.^[^ ^27^ ^]^ As illustrated in Figure 4f,h, the XA@Ag/H+US group demonstrated the most robust collagen synthesis, substantiating the notion that the synergistic application of a multifunctional hydrogel and ultrasound can markedly accelerate the wound healing cascade.

**Figure 6:**
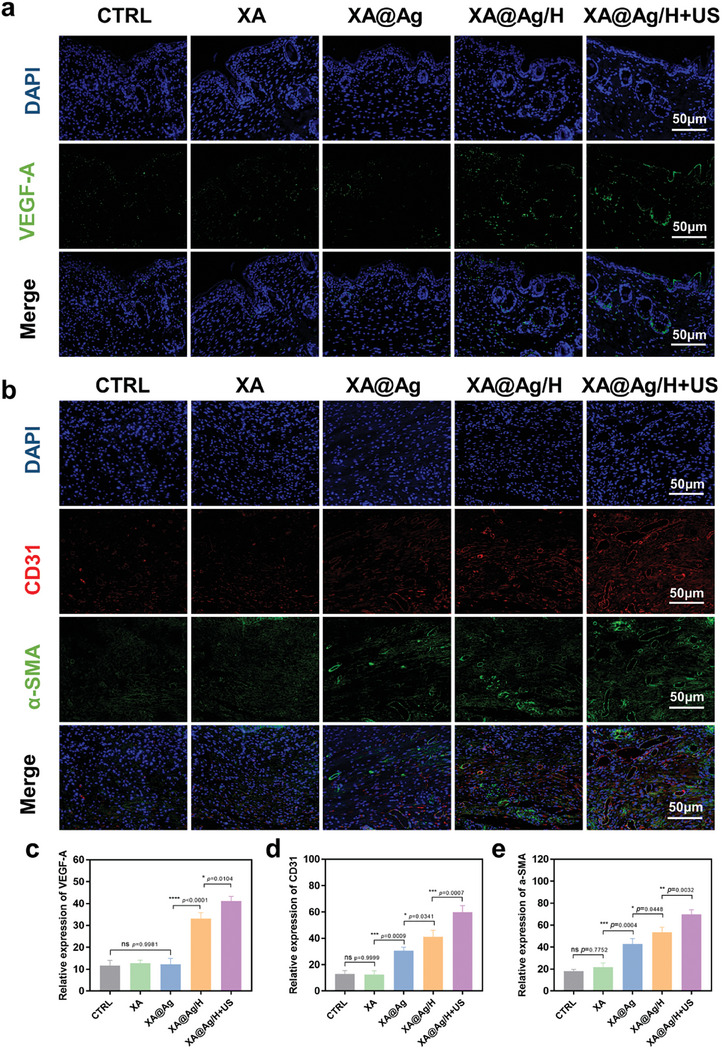
Immunofluorescence and immunohistochemistry of diabetic wound areas. a) Immunofluorescence of VEGF‐A in skin tissue of diabetic wounds on day 12. b) Immunofluorescence of a‐SMA and CD31 in diabetic wound skin tissue on day 12. c) Relative expression of VEGF‐A of diabetic wounds on day 12. d) Relative expression of CD31 in diabetic wound skin tissue on day 12. e) Relative expression of a‐SMA in diabetic wound skin tissue on day 12. Data represent mean ± SD; *p < 0.05, **p < 0.01, ***p < 0.001, ****p < 0.0001.

To elucidate the underlying mechanisms through which XA@Ag/H+US augments diabetic wound healing, an assessment of the hydrogel's angiogenic potential at the wound site was conducted. Utilizing immunofluorescence staining, the expression profiles of vascular endothelial growth factor (VEGF)‐A, alpha‐smooth muscle actin (α‐SMA), and cluster of differentiation 31 (CD31) within the wound tissue were scrutinized on day 12 post‐treatment with either phosphate‐buffered saline (PBS) or the disparate hydrogels (**Figure** **6**a,b). Notably, VEGF‐A expression was markedly upregulated in wounds treated with XA@Ag/H, as compared to those treated with XA or XA@Ag alone, suggesting a contributory role of the hydrogel's HBD peptide in angiogenesis (Figure 6c). Concurrently, the immunofluorescence intensity of α‐SMA and CD31, markers indicative of vascular smooth muscle and endothelial cells respectively, were employed to appraise the hydrogel's capacity to stimulate neovascularization. The XA@Ag/H group exhibited heightened fluorescence intensities compared to the XA@Ag group, reinforcing the notion that the hydrogel's peptide constituents are instrumental in angiogenic processes. Moreover, the XA@Ag/H+US treated wounds showcased pronounced neovascularization, characterized by the formation of new blood vessels and elevated expression levels of α‐SMA and CD31, surpassing those observed in the XA@Ag/H group (Figure 6d,e). This enhanced angiogenic response may stem from the ultrasonic disruption of biofilm architecture, permitting deeper infiltration of the hydrogel and its HBD peptide‐laden nanoparticles into the wound bed. Conversely, in the absence of ultrasonic treatment, the hydrogel faced impediments in achieving profound tissue penetration, thereby attenuating its angiogenic efficacy.

### 2.7 Assessment of Staphylococcus Aureus Infected Diabetic Wound Healing

Prior to animal experiments, to further study the thermal effect induced by ultrasound, thermal imaging camera was utilized to quantify the temperature variation. In detail, the hydrogel was injected onto the mice followed by ultrasound treatments of different durations: 0 min, 2 min, 5 min, 10 min. As shown in Figure S12 (Supporting Information), after ultrasound stimulation (15 W) for 10 min, the mouse body temperature increased to 36.8°C, which is slightly above the normal body temperature of mice. To further evaluate the possible damage to skin tissue, HE staining of mouse skin after ultrasound was imaged. As shown in Figure S13 (Supporting Information), no obvious damage of mouse skin and an intact epidermis and appendages were observed.

**Figure 7:**
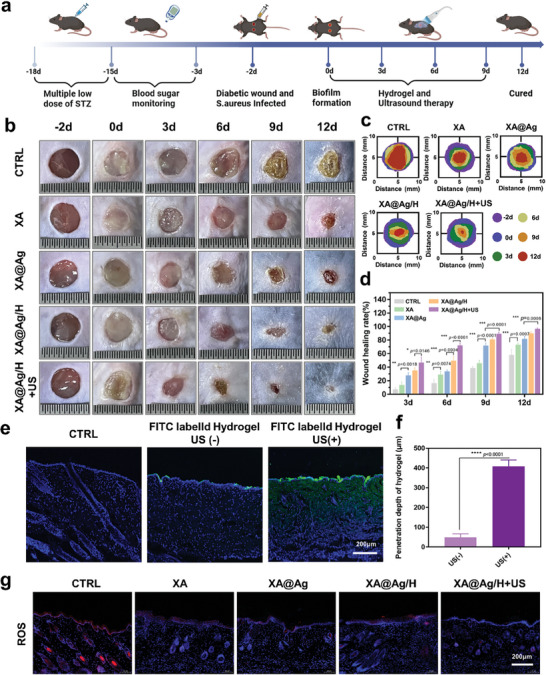
Evaluation of biofilm infected diabetic wound healing. a) Schematic diagram of the construction of infected diabetic wound model and Ultrasound‐responsive hydrogel treatment programs. b) Photographs of different treatments of diabetic wounds infected with biofilm at −2, 0, 3, 6, 9, and 12 days. c) Traces of wound closure during 12 days. d) Relative wound close rate calculated based on corresponding digital images. e) and f) Delivery depth of ultrasonic responsive hydrogel in skin wound. g) ROS fluorescent staining of wound area on day 12. Data represent mean ± SD; *p < 0.05, **p < 0.01, ***p < 0.001, ****p < 0.0001.

Expanding upon the in vitro findings regarding biofilm eradication, we advanced to the establishment of a Staphylococcus aureus biofilm model on diabetic mouse wounds to evaluate the in vivo therapeutic efficacy of the ultrasound‐responsive HBD peptide hydrogels concerning their anti‐biofilm activity and reparative prowess (**Figure** **7**a). Post‐inoculation of the wounds with S. aureus, the manifestation of robust biofilms across all experimental groups was an observed phenomenon (Figure 7b). At the 3‐day mark, the wound area in both the blank and XA groups exhibited negligible reduction, with biofilms persisting conspicuously on the wound surfaces. Conversely, the XA@Ag and XA@Ag/H groups demonstrated a significant decrease in wound area, by 27.97% and 35.23% respectively, and a diminished biofilm presence in comparison to the control group. Notably, the group administered with ultrasound treatment showcased a pronounced reduction in biofilm on the third day, with a substantial decrease in wound area of 72.3% by the 6th day (Figure 7d). At the 12‐day mark, complete wound closure was observed, indicative of the ultrasound‐responsive HBD peptide hydrogel's efficacy in promoting healing in diabetic wounds with biofilm infection.

Additionally, wound samples collected on the 12th day were cryopreserved and analyzed for reactive oxygen species (ROS) levels (Figure 7g; Figure S14, Supporting Information). The cryosections were stained with a radical fluorescent probe (DHE) and examined under confocal laser scanning microscopy (CLSM). The control group, treated exclusively with phosphate‐buffered saline (PBS), displayed intense fluorescence, whereas the mice treated with the hydrogel groups (XA, XA@Ag, XA@Ag/H, and XA@Ag/H+US) exhibited markedly subdued fluorescence signals (Figure 7g). The hydrogel's antioxidant efficacy is predominantly attributable to the xanthan gum component, while the silver ions and HBD peptides, have demonstrated significant potency in bactericidal activity and angiogenesis promotion, thereby synergistically augmenting the wound healing process. After 12‐day therapeutic regimen involving distinct hydrogel formulations, tissue sections were procured from the murine cardiac, hepatic, splenic, pulmonary, and renal tissues. Histological examination, facilitated by hematoxylin and eosin (H&E) staining, revealed a remarkable absence of any discernible histological aberrations or pathological manifestations across the assessed organs. No obvious side effects were observed with hydrogel treatment (Figure S16, Supporting Information), indicating its potential as viable pharmaceutical candidates in clinic.

The majority of chronic diabetic wounds are encumbered by inflammatory exudates and biofilms, which erect a physical and chemical barrier that markedly diminishes the bioavailability of topical therapeutics. To highlight the advantages of ultrasound‐responsive hydrogels in the treatment of diabetic infected wounds, we employed FITC to label the hydrogels, thereby facilitating the observation of their distribution within skin wounds and enabling a comparative analysis of the therapeutic efficacy between hydrogels with and without ultrasound treatment. As illustrated in Figure 7e,f, hydrogels devoid of ultrasound treatment remained superficial and exhibit limited penetration into the wound bed (Figure S15, Supporting Information). Compared with the group without ultrasound treatment, hydrogels subjected to ultrasonic treatment effectively penetrate the skin, achieving deeper wound penetration, and exhibit enhanced therapeutic efficacy as the duration of ultrasound treatment increases. Traditional topical hydrogel delivery methods face limitations in addressing biofilm‐infected diabetic wounds. In contrast, the ultrasound‐activated hydrogel in this study has demonstrated the ability to surmount biological barriers, enabling more efficacious drug delivery, complete biofilm eradication, reduction of oxidative stress, and promotion of wound angiogenesis, all of which collaboratively contribute to the wound healing process.

## 3 Conclusion

In this study, an ultrasound‐responsive hydrogel incorporated with heparin‐binding domain (HBD) peptide nanoparticles was developed to achieve deep skin penetration for promoting diabetic chronic wound healing. Derived from von Willebrand Factor with angiogenic activity, HBD peptides were first engineered to self‐assemble into nanoparticles with enhanced biostability and bioavailability and then loaded within ultrasound‐responsive hydrogel for controlled delivery toward deep wound issue. External ultrasound stimulation could break the crosslinking to degrade hydrogel into smaller fragments and reduce interstitial pressure to promote deep wound penetration with ≈400 µm depth. In addition, desired antioxidant and antibacterial activity of such hydrogel was observed. Animal studies have demonstrated that HBD peptide nanoparticles loaded hydrogel under ultrasound sonication was able to effectively eliminate biofilms and achieve complete wound closure in diabetic mice with chronic wounds, indicating the effectiveness of this proposed anti‐biofilm strategy. Specifically, in vivo studies also showed that HBD peptide nanoparticles loaded hydrogel increased the levels of angiogenesis‐related growth factors (VEGF‐A, CD31, and α‐SMA) to effectively accelerate wound repair. Such experimental data revealed that the developed ultrasound‐responsive HBD peptide hydrogel provides a novel therapeutic approach to achieve efficient delivery toward deep wound site and eliminate biofilms to promote angiogenesis and diabetic wound healing.

## 4 Experimental Section

### Chemicals

Xanthan gum (XA) and Sodium alginate (SA) was purchased from Aladdin (Shanghai, China); HBD peptide was purchases from Anhui Zhuan tai Biotechnology Co., Ltd (Anhui, China); Calcium chloride (10043‐52‐4) was purchased from ChemicalBook (Shanghai, China); Human umbilical vein endothelial cells (HUVECs) and NIH/3T3 cells were acquired from the Gaining Biological (Shanghai, China);Staphylo‐coccus aureus strain (S. aureus, ATCC25923), Escherichia coli strain (E. coli, ATCC25922), were acquired from the ATCC. LB Nutrient Agar Plate was purchased from Henan Meikai Bio‐technology Co., Ltd (Henan, China). Calcein AM /PI Cell Double Stain Kit (MX3012) and SYTO 9/Pl Live/Dead Bacteria Double Stain kit (MX4234) were purchased from Shanghai Maokang Biotechnology Co., Ltd (Shanghai, China); Cell Counting Kit‐8 was purchased from Beyotime (Shanghai, China); a‐smooth Muscle Actin (A17910) and VEGF‐A antibodies (A23759) was purchased from Abclonal (Wuhan, China); CD31 antibody were obtained from Bioss (Beijing, China). Ultrasound therapy device (WED‐101) was purchased from Shenzhen Welld Medical Electronics Co., Ltd (Shenzhen, China).

### Cells Culture

NIH/3T3 cells from Shanghai Gaining Biotechnology Co., Ltd, were cultured in DMEM (Gibco, 11965092) with 10% fetal bovine serum and 1.0% antibiotics. HUVEC cells were cultured in Endothelial Cell Medium (Sciencell,1001) with 1% endothelial cell growth supplement (ECGS) and 1% Penicillin‐Streptomycin Solution and 10% fetal bovine serum.

### Culture of Bacteria and Biofilm

The culture of bacteria and their biofilms involved the initial cultivation of single colonies of S. aureus and E. coli in LB medium at a temperature of 37°C in a shaker. When the optical density (OD) value reaches ≈0.5, indicating a concentration of ≈1×10^8^ CFU mL^−1^, the bacteria that have been harvested are utilized for the purpose of biofilm formation. In order to cultivate the biofilm, a volume of 100 uL of S. aureus (at a concentration of 1×10^8^ CFU mL^−1^) was incubated in a 96‐well plate at a temperature of 37°C for a duration of 48 h. Subsequently, the culture medium was discarded and the unattached bacteria were gently washed away by performing three rinses with phosphate‐buffered saline (PBS). The biofilm can then be observed at the bottom of the well.

### Synthesis of HBD Peptide Nanoparticles

A novel stock solution of the HBD (FF‐K (2‐naphthaleneacetic acid)‐YIGLKDRKRPSELRRIASQVKYA) peptide was meticulously prepared by initially rehydrating the lyophilized peptide in a 1 M phosphate‐buffered saline (PBS) solution to a precise concentration of 5.0 mg mL^−1^ for each experimental setup. This concentration was meticulously chosen to prevent any premature aggregation of the peptide. Subsequently, the pH of the solution was carefully titrated to a neutral pH of 8.5 using a 1 M sodium hydroxide solution. Following the pH adjustment, the mixture was subjected to continuous agitation for an extended period of 12 h to facilitate the formation of self‐assembled HBD peptide nanoparticles. The resulting HBD peptide nanoparticles, post self‐assembly, were then lyophilized for long‐term storage to preserve their structural integrity and ensure reproducibility in subsequent experiments.

### Synthesis of Ultrasound‐Responsive Hydrogels Loaded with Peptide Nanoparticles

Xanthan gum and sodium alginate, each at a concentration of 0.75 wt.%, were introduced into ionized water and subsequently dissolved through a combination of gentle heating and mechanical agitation. Following this, a solution of silver nitrate (AgNO3) at a molar concentration of 50.0 mM and a suspension of freshly prepared HBD peptide nanoparticles at a concentration of 0.50 mg mL^−1^ were homogeneously pre‐mixed with the hydrogel solution. This mixture was then carefully aliquoted, with 1.0 mL of it being transferred into each well of a 12‐well tissue culture plate. Subsequently, 1.0 mL of a 0.1 M calcium chloride (CaCl_2_) solution was incrementally introduced along the periphery of the plate walls, allowing for a controlled interaction. The plate was then undisturbed for a period of 10 min to facilitate crosslinking. Post‐crosslinking, the peptide nanoparticle‐laden hydrogel was thoroughly rinsed with deionized water to remove any unreacted components and to ensure a pristine hydrogel matrix.

### Morphological Characterization of the HBD Peptide Nanoparticles Loaded Ultrasound Responsive Hydrogel

The TEM images morphology of the HBD peptide nanoparticles were taken with a JEOL JEM2100F instrument at 200 kV equipped with a Gatan 894 Ultrascan 1 k CCD camera. Hydrogels with different components were evaluated with scanning electron microscopy (Zeiss Sigma 300 VP instrument). The characteristics were noted twice using electronic mode at 2 kV.

### Modulus of the HBD Peptide Nanoparticles Loaded Ultrasound Responsive Hydrogel

Rheological characterization of the hydrogel, both prior to and following exposure to ultrasound, was executed utilizing a HAAKE rheometer. The initial gap set between the rheometer's parallel plate fixtures was standardized at 5 mm to ensure consistency across all tests. A frequency sweep test was implemented, spanning a frequency spectrum of 1 to 10 Hz, while maintaining a constant shear strain of 1%. This was complemented by a strain sweep test, which varied the strain from 0.1% to 30% at a constant angular frequency of 1 rad s^−1^. The objective of these sweeps was to ascertain the storage modulus (G′) and loss modulus (G″) values, which are indicative of the hydrogel's elastic and viscous responses, respectively. To ensure the reliability of the findings, each test was performed on distinct hydrogel samples, serving as replicates, thereby allowing for the assessment of variability and the robustness of the rheological measurements.

### Cytocompatibility and Hemocompatibility Assessment

The biocompatibility of various hydrogels (XA, XA@Ag, XA@Ag/H) with cells was evaluated using the CCK‐8 assay kit (Beyotime Bioengineering Co., Ltd, China). Initially, the hydrogels were prepared at a range of concentrations (12.5 µg mL^−1^, 25 µg mL^−1^, 50 µg mL^−1^, 100 µg mL^−1^, 200 µg mL^−1^, and 400 µg mL^−1^) using Dulbecco's Modified Eagle Medium (DMEM) and Endothelial Cell Medium (ECM) for cell culture, and stored at 4°C for future use. NIH/3T3 fibroblasts and Human Umbilical Vein Endothelial Cells (HUVECs) were suspended and evenly seeded into 96‐well plates at a density of 7500 cells per well. After allowing the cells to attach, the medium was refreshed the following day, and 20 µL of the hydrogel solution at varying concentrations was added to each well. The cells were then co‐incubated with the hydrogels at 37°C in an incubator for 24 h. Subsequently, serum‐free CCK‐8 reagent was added to the cells and incubated for an additional 2 h at 37°C. The absorbance at 450 nm was measured using a microplate reader, and cell viability was calculated using the formula:

(1) $$\begin{array}{ccc} Cell\;viability\left(\%\right) & = & \left[OD_{450}\left(sample\;group\right)-OD_{450}\left(blank\;group\right)\right]/\\ & & \left[OD_{450}\left(control\;group\right)-OD_{450}\left(blank\;group\right)\right]\;\times 100\% \end{array}$$

Furthermore, the cytotoxicity of the hydrogels toward cells was assessed using the Calcein AM/PI Double Stain Kit (Maokangbio, MX3012). NIH/3T3 cells were plated in a 24‐well plate and cultured for 24 h. Following this, hydrogels at a concentration of 200 µg mL^−1^ were added to the cells according to their respective groups. After a further 48‐hour incubation period, the cells were incubated with the Calcein/PI working solution for 30 min. The cells were then rinsed with Phosphate‐Buffered Saline (PBS) and observed using a fluorescence microscope (Olympus, Japan) to evaluate cell viability and cytotoxicity.

### Hemocompatibility of Hydrogels In Vitro

The hemocompatibility of the hydrogel was evaluated using an in vitro hemolysis assay. Fresh mouse blood (2 mL) was collected into an anticoagulant‐containing tube. After centrifugation at 3000 rpm for 10 min, the plasma supernatant was discarded, and the erythrocytes (RBCs) were washed three times with 0.9% sodium chloride (NaCl) solution to obtain a purified RBC suspension. An aliquot of 100 µL of the purified RBCs was transferred into a 1.5 mL centrifuge tube, followed by the addition of 100 µL of the test solutions: deionized water, phosphate‐buffered saline (PBS), XA hydrogel, XA@Ag hydrogel, and XA@Ag/H hydrogel. The volume in each tube was adjusted to 1 mL with 0.9% NaCl, mixed thoroughly, and incubated at 37°C for 3 h. Deionized water served as the positive control, inducing complete hemolysis, while PBS was the negative control with no induced hemolysis. After the incubation period, the tubes were centrifuged at 3000 rpm for 10 min. The RBC mixture in the tubes was photographed, and 100 µL of the supernatant was collected. The absorbance was measured at a wavelength of 545 nm (n = 5) using a spectrophotometer. The hemolysis rate (HR%) was calculated using the following formula:

(2) $$Hemolysis\;Rate\;\left(\%\right)=\left[\left(Ah-An\right)/\left(At-An\right)\right]\times 100\%$$

Among them, Ah, At, and An represent the absorbance at 545nm of the sample, positive control and negative control respectively.

### Cell Proliferation and HUVEC Migration Assays

The cell proliferation induced by the hydrogels was assessed using the CCK‐8 assay kit. NIH/3T3 fibroblasts, exhibiting good morphology, were seeded at a density of 5000 cells per well in a 96‐well plate. Once the cells had adhered, the original growth medium was aspirated, and the wells were replenished with Dulbecco's Modified Eagle Medium (DMEM) containing hydrogel at a final concentration of 50 µg mL^−1^. The cells were then co‐incubated with the hydrogel at 37°C for 24 and 72 h, respectively. Post‐incubation, the CCK‐8 assay was employed to measure the relative cell viability. Furthermore, the effect of the hydrogel on the proliferation of Human Umbilical Vein Endothelial Cells (HUVECs) was evaluated by fluorescence staining. A suspension of HUVECs was prepared at a lower concentration and evenly distributed in a 24‐well plate. After allowing the cells to adhere, the culture medium was replaced with an Extracellular Matrix (ECM) medium containing hydrogel at a concentration of 50 µg mL^−1^ and incubated for 24 h. Following a 72‐hour incubation period, the medium was removed, and the cells were washed three times with Phosphate‐Buffered Saline (PBS). A staining solution (250 µL) was added, and the cells were incubated at 37°C for 15 min. The staining solution was then aspirated, the cells were washed three times with PBS, and the cell density was observed under a fluorescence microscope.

### Evaluation of Hydrogels for Promoting HUVEC Migration

HUVECs in an optimal growth condition were collected and prepared as a cell suspension. The suspension was evenly distributed across 6‐well plates and incubated overnight at 37°C in a humidified incubator. Once a uniform monolayer of cells had formed, a 20 µL pipette tip was used to create controlled scratches (wounds) in the cell monolayer. The detached cells from the scratches were gently washed away by rinsing with PBS three times. Subsequently, 2 mL of ECM medium devoid of Fetal Bovine Serum (FBS) and containing hydrogels XA, XA@Ag, and XA@Ag/H at a concentration of 50 µg mL^−1^ was added to each well, and the cells were co‐incubated. The wells were observed under a microscope at 0 h and 24 h to capture images and measure the width of the wound. Before each photographic capture, the floating cells were effectively removed by rinsing thoroughly with PBS. The wound closure rate at 24 h was calculated using the following formula:

(3) $$Wound\;closure\;rate\;\left(\%\right)=\left[\left(W_{0h}-W_{24h}\right)/W_{0h}\right]\times 100\%$$

### In Vitro Antioxidant Efficacy

The antioxidant capabilities of the XA hydrogel were assessed using a DPPH (2,2‐diphenyl‐1‐picrylhydrazyl) radical scavenging assay, as per the instructions provided with a commercial reagent kit (Beijing Solarbio Science & Technology Co., Ltd, China). Additionally, the XA, XA@Ag, and XA@Ag/H hydrogels were evaluated for their hydrogen peroxide (H_2_O_2_) scavenging activity. For this, the hydrogels were incubated in 2.0 mL of phosphate‐buffered saline (PBS) containing 200 µM H_2_O_2_ at 37°C for 24 h. The residual H_2_O_2_ concentration was then quantified using a Hydrogen Peroxide Detection Kit (Beijing Solarbio Science & Technology Co., Ltd, China) by measuring the absorbance at 405 nm. The H_2_O_2_‐eliminating capacity of the hydrogels was calculated from the absorbance data.

### In Vitro Procoagulant Capacity Test and In Vivo Liver Hemostatic Ability Test

*In Vitro Procoagulant Ability Study*: Fresh mouse blood samples were collected and immediately transferred into tubes containing anticoagulant, then stored at 4°C for subsequent analysis. Distinct hydrogels, each weighing 200 mg, were placed at the base of a 96‐well plate. To each well, 100 µL of fresh anticoagulated blood was added, ensuring thorough mixing with the hydrogel. The mixture was incubated for varying durations ranging from 1 to 8 min. At selected time points, the non‐coagulated blood was gently aspirated and washed three times with physiological saline. The coagulated blood was then photographed using a digital camera to evaluate the in vitro procoagulant properties of the hydrogels.

*In Vivo Hemostatic Studies*: The in vivo hemostasis experiments were conducted using a mouse liver bleeding model. Animals were randomly assigned to four groups: a blank control, an XA group, an XA@Ag group, and an XA@Ag/H group, with three mice per group. Mice were anesthetized, their abdominal fur was removed, and the skin was disinfected with iodine. The chest cavity was opened to expose the liver. A weighed piece of filter paper was placed beneath the liver to collect any blood loss. The operating table was tilted to an angle of 30–45 degrees. A 6 mm hole was created in the liver using a standardized punch device to induce bleeding. The liver injury was immediately treated with the respective hydrogel blocks. Hemostatic time and blood loss volume were measured to assess the hydrogel's efficacy in controlling hemorrhage in the mouse liver model.

### In Vitro Antimicrobial Activity and Anti‐Biofilms Assay

Bacterial strains Staphylococcus aureus and Escherichia coli, each at a concentration of 10^8^ colony‐forming units per milliliter (CFU mL^−1^), were subjected to a 10 000‐fold dilution. Following this, 500 µL of the diluted bacterial suspension was mixed with 200 µL of the respective hydrogel and incubated at 37°C for 60 min. For the ultrasound treatment group, the mixture was exposed to ultrasound for 10 min. After incubation, 30 µL of the bacterial‐hydrogel mixture was evenly spread onto agar plates, which were then incubated at 37°C for 24 h. The resulting bacterial colonies were counted to determine the colony‐forming units.

Various hydrogels were pre‐incubated with Luria‐Bertani (LB) broth for 24 h. Post‐incubation, equal volumes of S. aureus and E. coli were added to the supernatants, and the mixtures were incubated in a 96‐well microplate for an additional 48 hours. The optical density (OD) at a wavelength of 600 nm was measured at regular intervals to monitor bacterial growth.

Scanning electron microscopy (SEM) was utilized to assess bacterial morphology post‐treatment. Bacterial suspensions that had undergone different treatments were spread onto coverslips. The samples were then fixed with a 4% paraformaldehyde solution for 30 minutes and dehydrated using a graded ethanol series (25%, 50%, 75%, 100%). Finally, prior to SEM examination, the samples were sputter‐coated with a thin layer of gold.

The remaining biofilm biomass across different experimental groups was evaluated using the crystal violet assay. After exposure to various hydrogels, the biofilms were air‐dried in a fume hood for 20 min. Each well was then stained with 500 µL of a 0.1% crystal violet solution for 15 min. Post‐staining, the crystal violet was aspirated off, and the wells were rinsed three times with sterile saline to remove any unbound dye. The bound crystal violet was solubilized by adding 500 µL of 95% ethanol to each well, and the absorbance was measured at a wavelength of 595 nm using a spectrophotometer.

The biofilms formed on the coverslips were analyzed for viability and cell death using the LIVE/DEAD staining kit (Invitrogen, USA). Following the staining protocol, the fluorescence intensity and the architectural complexity of the biofilms were assessed using a confocal laser scanning microscope (Olympus FV3000, Japan). The fluorescence microscopy allowed for the quantification of both live (SYTO9‐stained) and dead (propidium iodide‐stained) bacterial cells within the biofilm matrix.

### Evaluation of Ultrasound‐Enabled Deep Delivery of Hydrogels

To investigate the synergistic effect of ultrasound in facilitating the deep penetration of hydrogels, FITC (fluorescein isothiocyanate) was first incorporated into the hydrogel matrix. Subsequently, the FITC‐loaded hydrogel was applied topically to the wounded skin of mice. Ultrasound treatment was then administered for varying durations: 0 min, 5 min, 10 min, and 20 min. After a one‐hour incubation period, the hydrogel was carefully removed. The treated skin tissue was rapidly cryopreserved by immersion in liquid nitrogen to preserve the spatial distribution of the hydrogel components. Following cryopreservation, the skin tissue was sectioned into thin slices using a cryostat. The sections were then stained and examined under a confocal laser scanning microscope to visualize and measure the depth of hydrogel penetration into the skin tissue.

### Diabetic Wound Healing Assessment

All in vivo animal experiments were conducted according to the institutional guidelines and relevant regulations for Animal Experimentation of Laboratory Animals of Tongji University, and all animal experiments were approved by the Tongji University Animal Center's Ethics Committee (TJAF00224101). Obtain 7‐week‐old male C57BL/6J mice and acclimatize them to a temperature‐controlled vivarium for a week prior to experimentation. Prepare a streptozotocin (STZ) solution by dissolving it in a citric acid buffer (0.1M, pH 4.2–4.5) to a final concentration of 10 mg/mL, and store the solution in a dark environment to prevent degradation. Administer an intraperitoneal injection of STZ to all mice in a fasted state at a dosage of 70 mg kg^−1^ for 4 consecutive days. Commence continuous monitoring of the mice's blood glucose levels two weeks post‐injection. A fasting blood glucose concentration of ≥16.7 mmol mL^−1^ is indicative of diabetes mellitus. Subsequently, the mice should be randomly assigned into five experimental groups, totaling 30 mice. Anesthetize the mice, and perform depilation and sterilization procedures prior to creating a full‐thickness skin wound with a diameter of 6 mm. Apply different hydrogels onto the wound surface and secure the area with a sterile adhesive film. Document the wound's morphology on days 0, 3, 6, 9, and 12 post‐wounding. The percentage of wound closure can be determined using the following formula:

(4) $$\begin{array}{ccc} Wound\;closure\;\left(\%\right) & = & \left(Initial\;wound\;area-Final\;wound\;area\right)/\\ & & Initial\;wound\;area\times 100 \end{array}$$

To systematically assess the wound healing process, euthanize mice in staggered intervals on days 6 and 12. Collect the skin tissues for histological evaluation, including hematoxylin and eosin (HE) staining, Masson's trichrome staining, and immunofluorescence analysis.

### Immunofluorescence

In order to assess the impact of HBD peptide hydrogel on enhancing neovascularization in diabetic wounds, the levels of various factors (CD31, α‐SMA, and VEGF‐A) were analyzed by immunofluorescence, as described in the following experimental procedure:(1)Skin tissue was taken from the wound area of mice on day 12 and fixed in 4% paraformaldehyde;(2)Paraffin slices, bake the slices to dry before placing them at 65°C for more than 4h.(3)Paraffin slice dewaxing to water: sequentially put the slice into environmental protection dewaxing liquid I 10min – environmental protection dewaxing liquid II 10min – environmental protection dewaxing liquid III 10min – anhydrous ethanol I 5min – anhydrous ethanol II 5min – distilled water washing.(4)Antigen repair: After repair is completed, cool naturally. Wash the slides in PBS (pH 7.4) on a decolorizing shaker by shaking 3 times for 5 min each time.(5)Circle serum closure: After the sections are slightly dried, draw a circle around the tissue with a histochemical pen and close it with a drop of BSA for 30 min.(6)Addition of primary antibodies: add the prepared primary antibodies (VEGF‐A, CD31 and a‐SMA) dropwise and incubate the sections flat in a wet box at 4°C overnight.(7)Addition of secondary antibody: The slides were washed three times in PBS (PH7.4) on a decolorizing shaker with shaking for 5 min each time, and the corresponding secondary antibody was added and incubated for 50 min at room temperature, protected from light.(8)DAPI re‐staining of nuclei: the slides were washed in PBS (pH 7.4) on a decolorizing shaker with shaking for 3 times, each time for 5 min. DAPI staining solution was added and the slides were incubated for 10 min at room temperature, protected from light.(9)Quenching of tissue autofluorescence: slides were washed three times in PBS (pH 7.4) on a decolorizing shaker for 5 min each time. autofluorescence quencher solution was added for 5 min, and the slides were rinsed under running water for 10 min. (10) Sealer: anti‐fluorescence quenching sealer. (11) Image acquisition: Fluorescence images were acquired using an orthogonal fluorescence microscope (Nikon ECLIPSE C1).

### Statistical Analysis

All data are expressed as the mean ± standard deviation (SD). To perform the statistical analysis, GraphPad Prism 9.0 was used. Unpaired Student's t‐tests or one‐way analysis of variance were used for group comparisons. *p* values that were less than 0.05 were regarded as statistically significant. (*p < 0.05, **p < 0.01, ***p < 0.001, ****p < 0.0001).

## Conflict of Interest

The authors declare no conflict of interest.

## Supporting information



Supporting Information

## Data Availability

The data that support the findings of this study are available from the corresponding author upon reasonable request.
