# Supplementary material for: Ultrasound‐Responsive HBD Peptide Hydrogel with Antibiofilm Capability for Fast Diabetic Wound Healing
Source: Adv Sci (Weinh). 2024 Sep 9;11(42):2406022. doi: 10.1002/advs.202406022 (PMC11558141; doi:10.1002/advs.202406022)
Supplement: Supplementary file 1 — Supporting Information [file ADVS-11-2406022-s001.pdf]

ADVANCED SCIENCE
Open Access

Supporting Information

for *Adv. Sci.*, DOI 10.1002/advs.202406022

Ultrasound-Responsive HBD Peptide Hydrogel with Antibiofilm Capability for Fast Diabetic Wound Healing

Lanlan Zong, Runxin Teng, Huiqi Zhang, Wenshang Liu, Yu Feng, Zhengmao Lu, Yuxiao Zhou, Zhen Fan*, Meng Li* and Xiaohui Pu**

Supporting Information

Ultrasound-responsive HBD peptide Hydrogel with Antibiofilm Capability for Fast Diabetic Wound Healing

Lanlan Zong[1#], Runxin Teng[4#], Huiqi Zhang[1#], Wenshang Liu[1,2], Yu Feng[1], Zhengmao Lu[5], Yuxiao Zhou[3*], Zhen Fan[3,4*], Meng Li[2*], and Xiaohui Pu[1*]

1. State Key Laboratory of Antiviral Drugs, Henan Province Engineering Research Center of High Value Utilization to Natural Medical Resource in Yellow River Basin, School of Pharmacy, Henan University, N. Jinming Ave., Kaifeng 475004, China.
2. Department of Dermatology, Shanghai Children's Medical Center, Shanghai Jiaotong University School of Medicine, Shanghai 200127, People's Republic of China
3. Department of Gynaecology and Obstetrics, Shanghai Key Laboratory of Anesthesiology and Brain Functional Modulation, Clinical Research Center for Anesthesiology and Perioperative Medicine, Translational Research Institute of Brain and Brain-Like Intelligence, Shanghai Fourth People's Hospital, School of Medicine, Tongji University, Shanghai 200434, China
4. Department of Polymeric Materials, School of Materials Science and Engineering, Tongji University, 4800 Caoan Road, Shanghai 201804, China.
5. Department of Gastrointestinal Surgery, The First Affiliated Hospital of Naval Medical University, Shanghai 200433, People's Republic of China

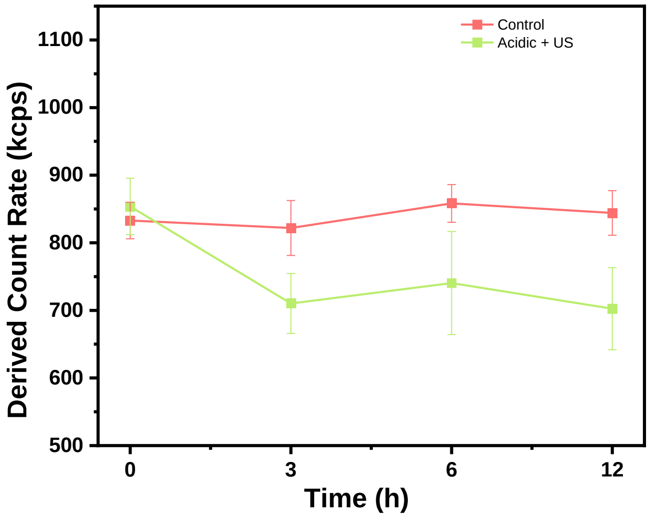

Figure S1. Derived count rate of HBD nanoparticles without ultrasound under neutral environment and with ultrasound under acidic environment to mimic wound site. (n=3 independent samples, mean ± SD).

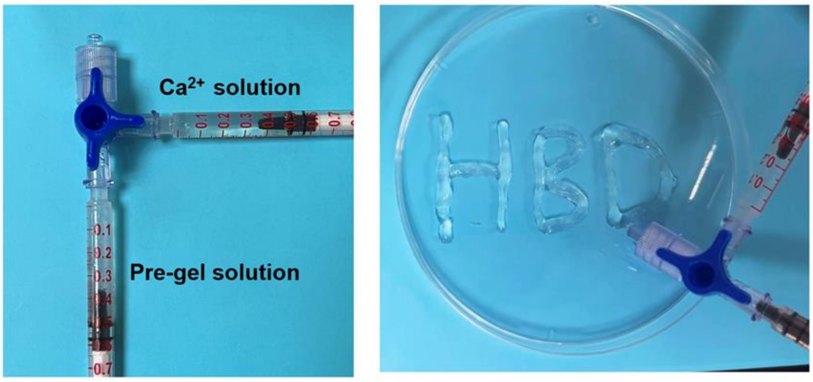

Figure S2. Injectable hydrogels synthesized through two syringes connected with a three-way stopcock.

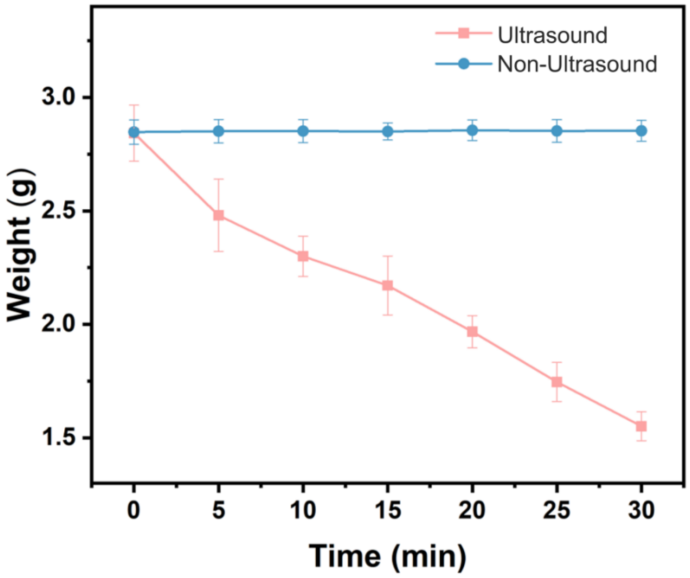

Figure S3. Ultrasound-responsive degradation of hydrogels. Weight changes of hydrogels after ultrasound and non-ultrasound. (n=3 independent samples, mean ± SD).

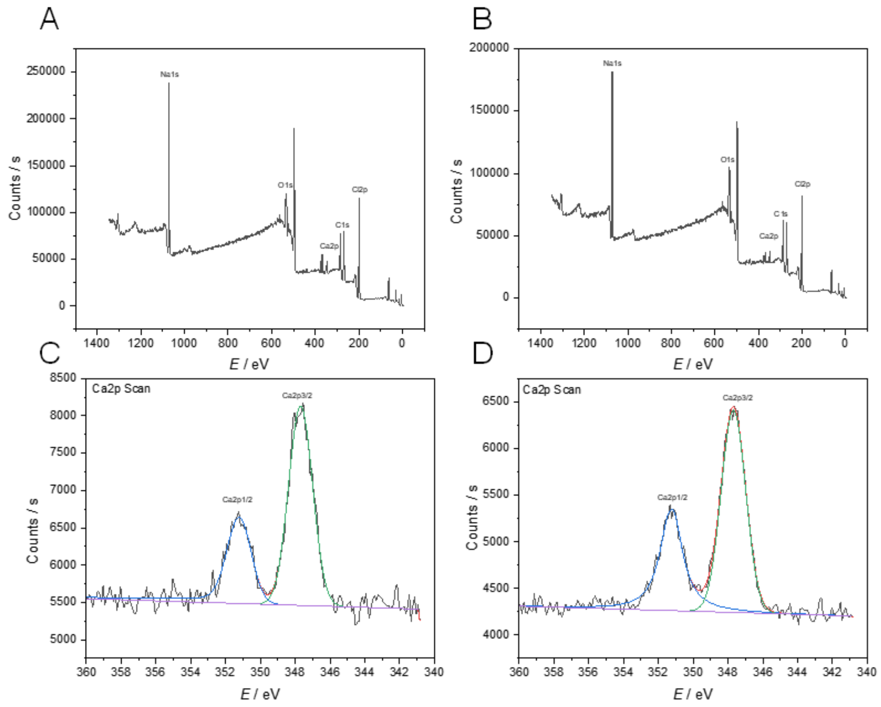

Figure S4. The XPS spectra of Xanthan gum hydrogel (A) before and (B) after ultrasound stimulation. XPS Ca2p narrow scans with the curve fit of Xanthan gum hydrogel (C) before and (D) after ultrasound stimulation.

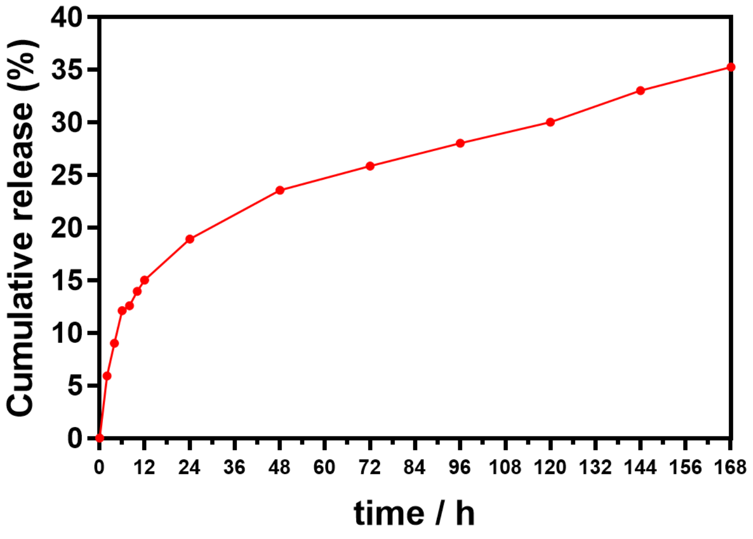

Figure S5. Release profile of hydrogel without ultrasound within one week.

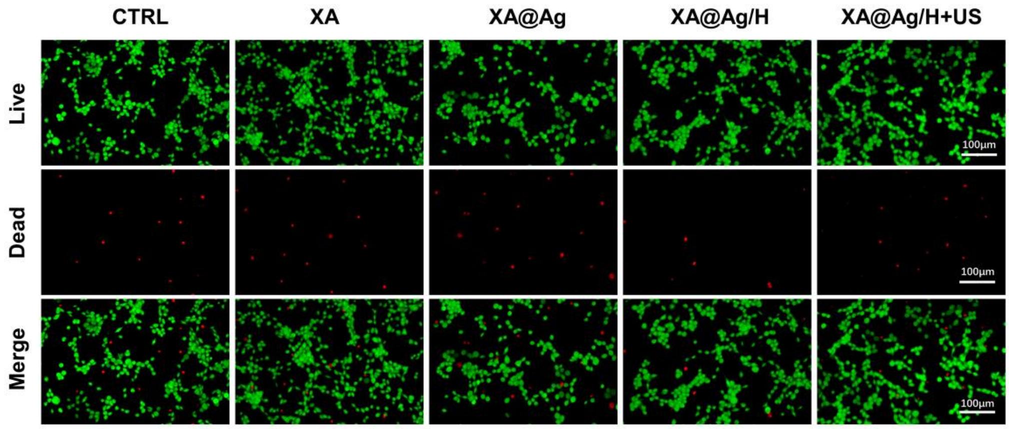

Figure S6. Live/Dead staining of NIH/3T3 cells after incubation with different hydrogels（500ug/ml） for 48 hours. Two independent experiments were performed and representative results are shown.

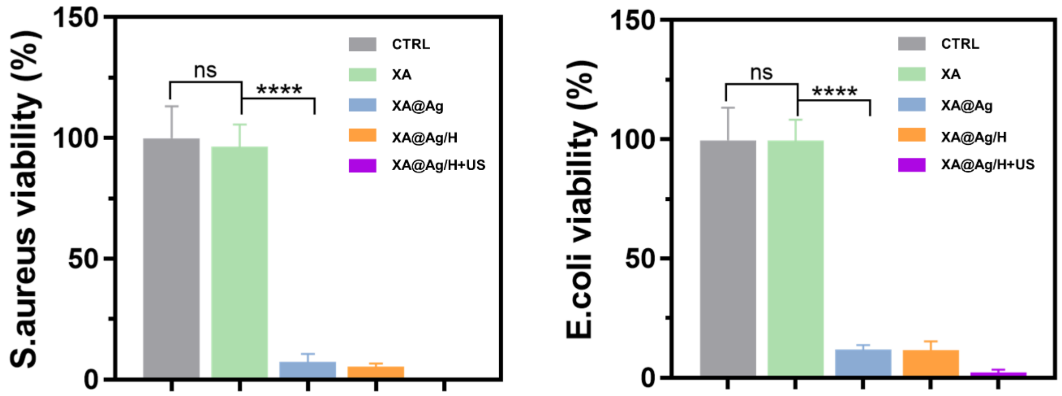

Figure S7. Relative bacterial viability calculated based on the number of colonies on agar plates. (n=3 independent samples, mean ± SD). ****p< 0.0001.

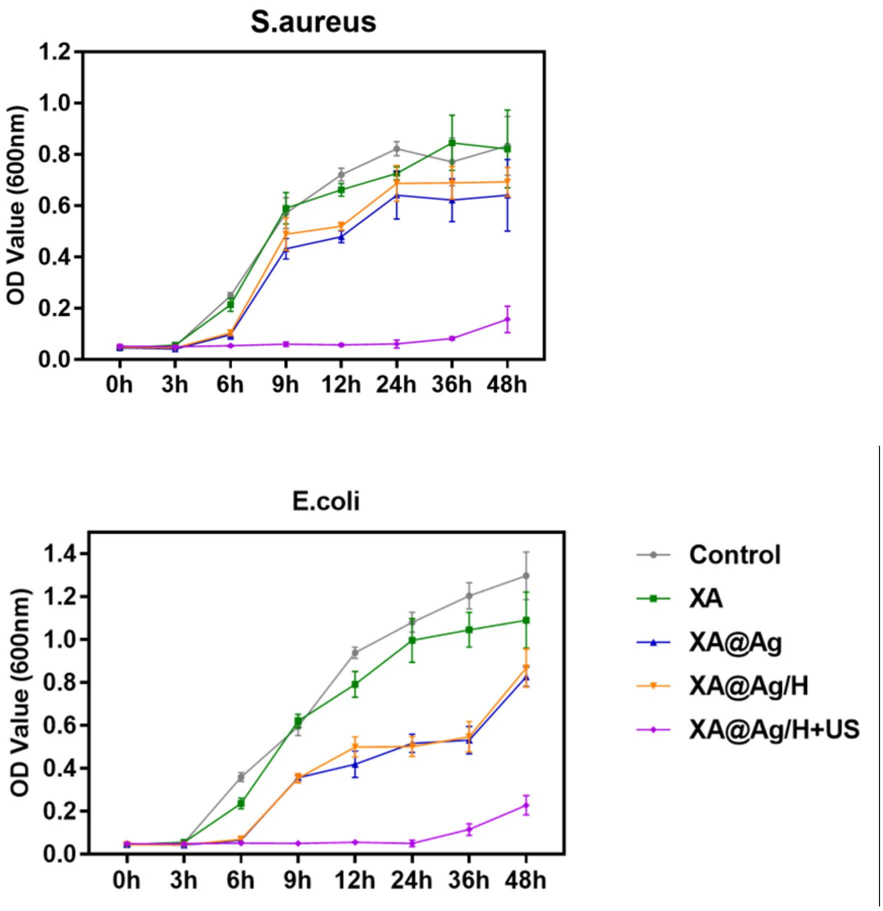

Figure S8. Growth curve of Staphylococcus aureus and Escherichia coli incubated with different hydrogels. (n=3 independent samples, mean ± SD).

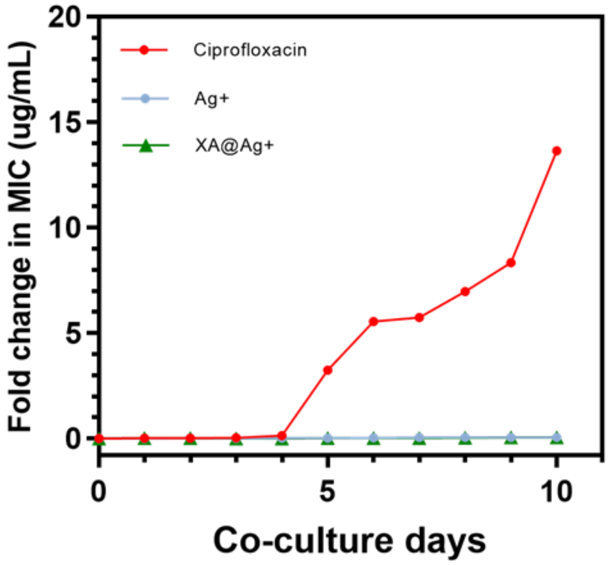

Figure S9. MIC values of ciprofloxacin, silver ions aqueous solution and silver ions loaded hydrogel against S. aureus (ATCC 25923) after exposing bacteria at their sub-MIC for 10 consecutive days.

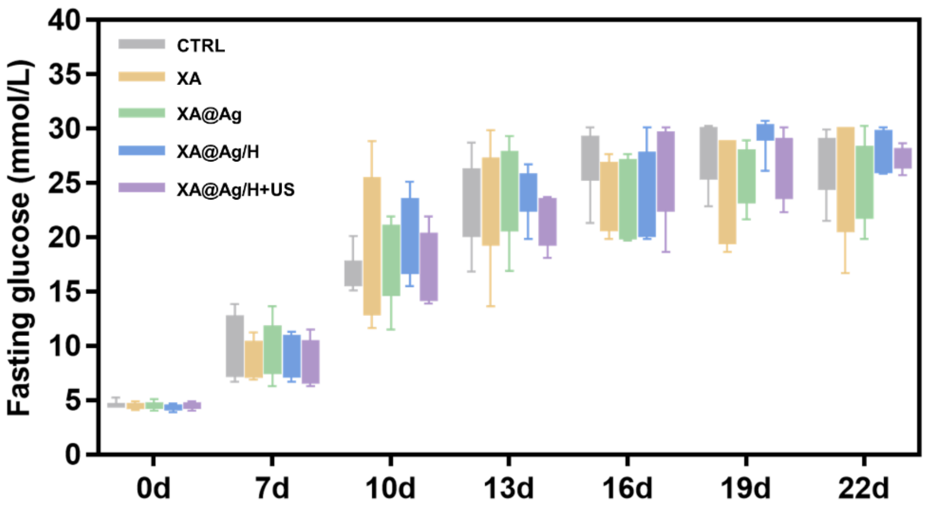

Figure S10. The fluctuation process of fasting blood sugar in mice after STZ injection for four consecutive days. (n = 3 independent samples, mean ± SD).

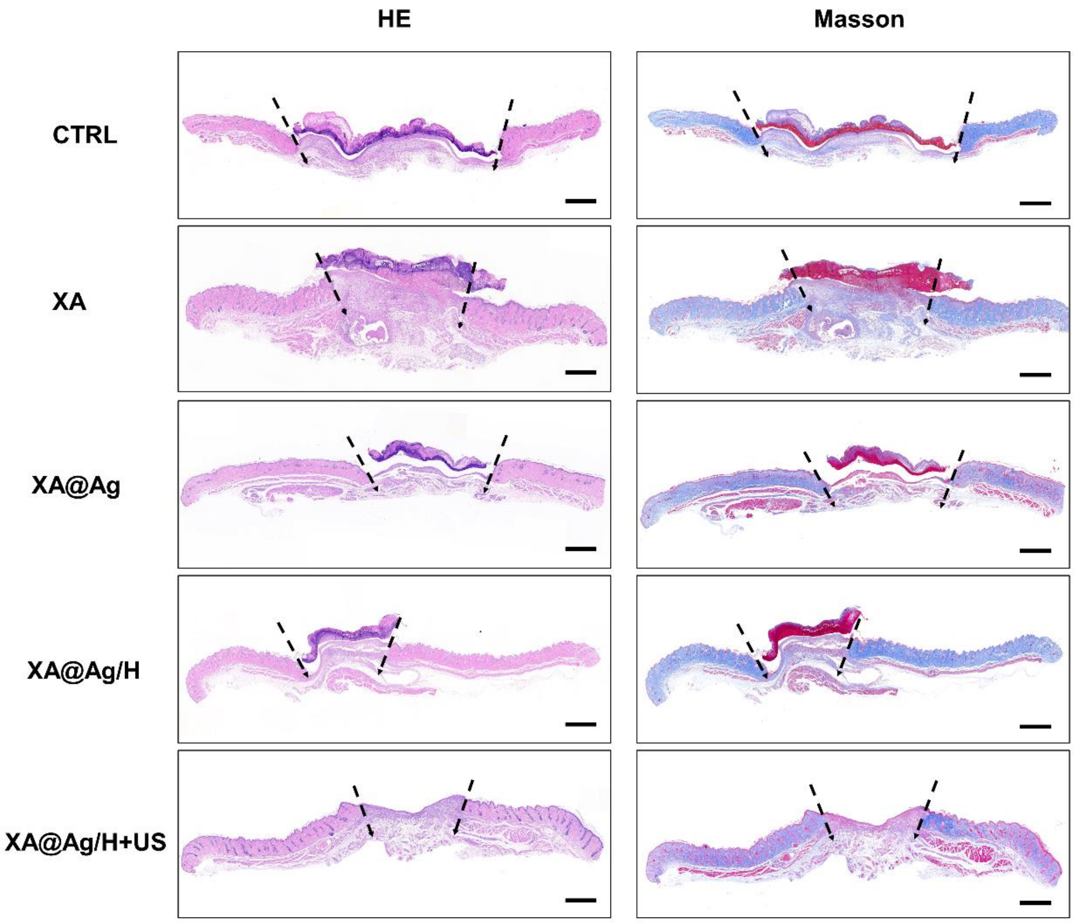

Figure S11. Hematoxylin and eosin-stained (H&E) and Masson's trichrome staining of skin tissue in mouse wound area on day 6.Scale bar=750μm. (n = 3 independent samples, mean ± SD).

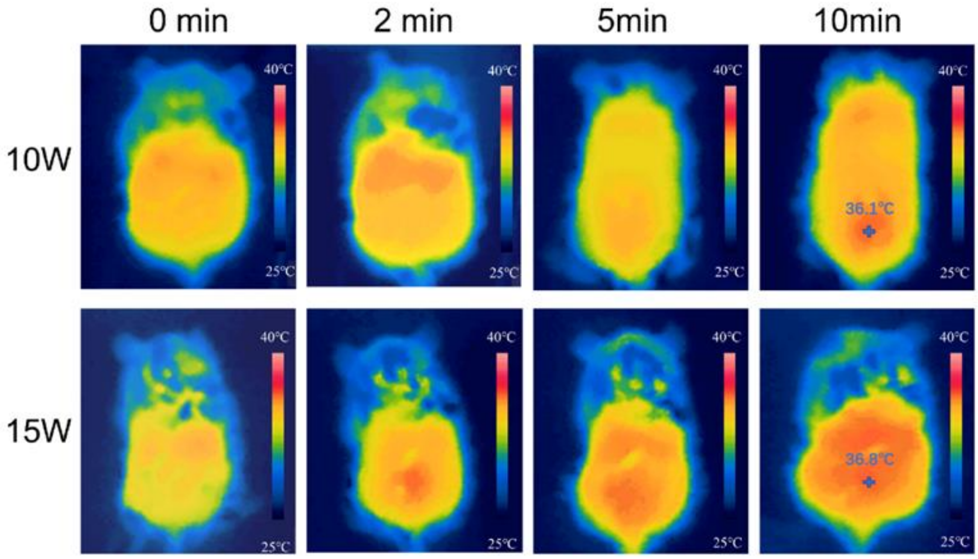

Figure S12. Thermal images of mouse skin with ultrasound stimulation of different power and time.

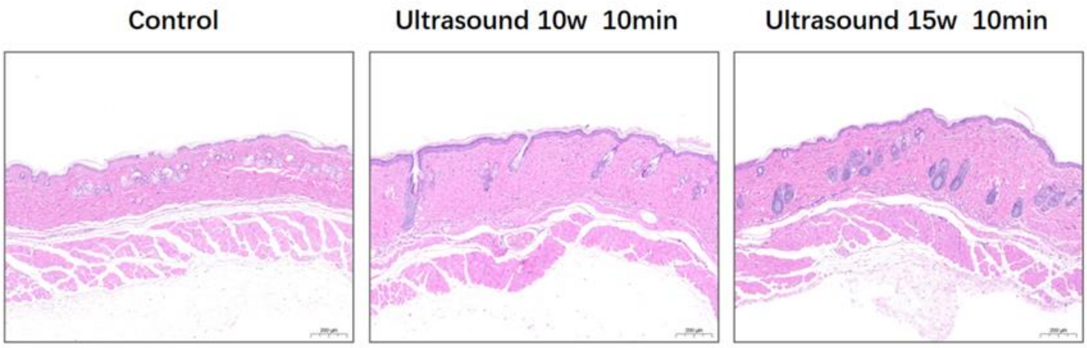

Figure S13. HE staining of mouse skin after ultrasound stimulation.

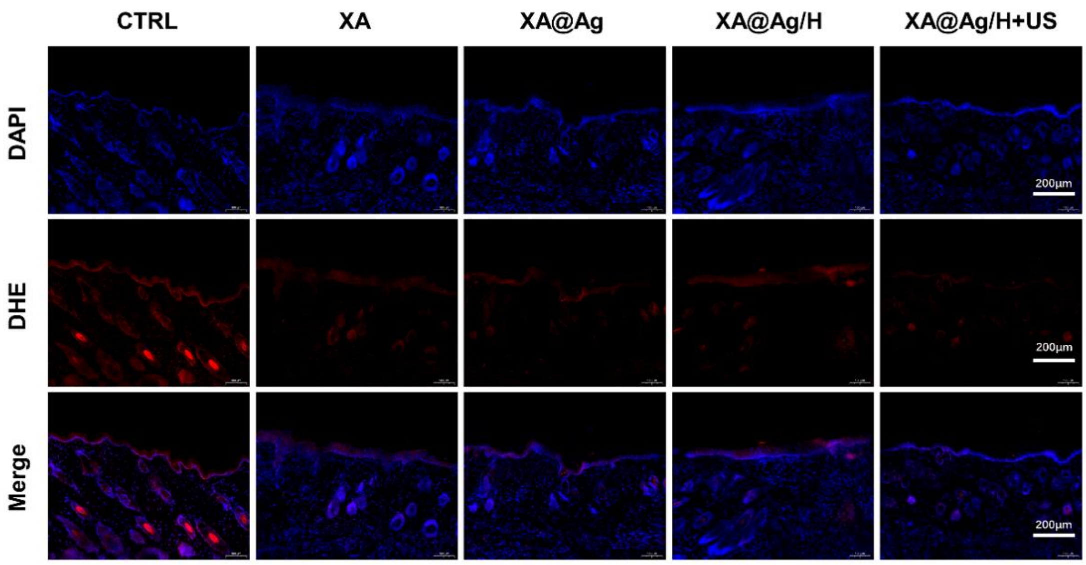

Figure S14. Immunofluorescence staining of ROS in the wounded region.

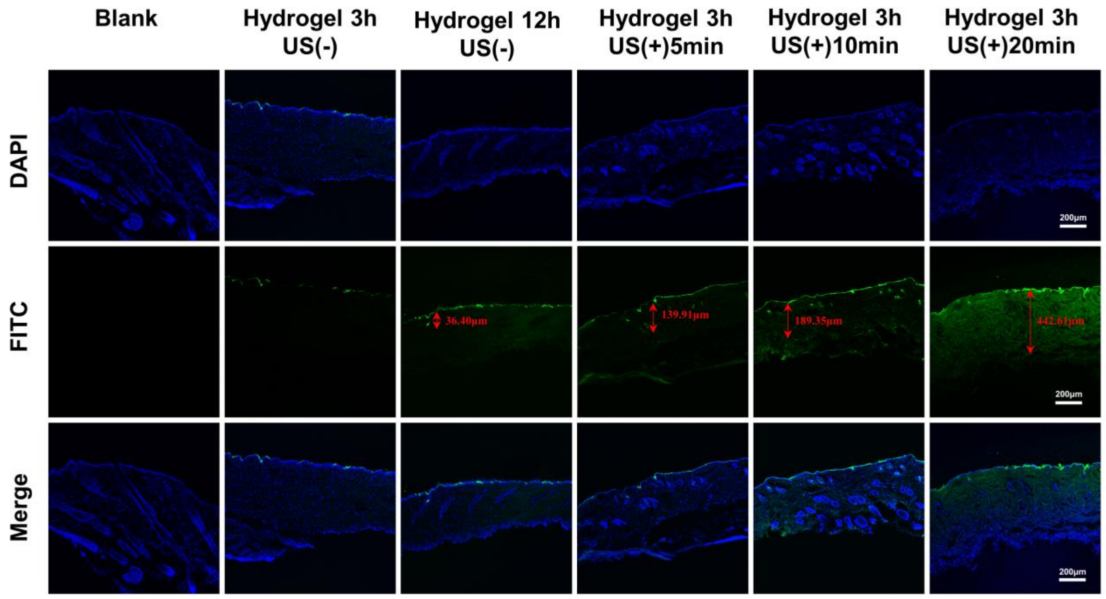

Figure S15. Ultrasound promotes the penetration of hydrogel into skin tissue. Three independent experiments were performed and representative results are shown.

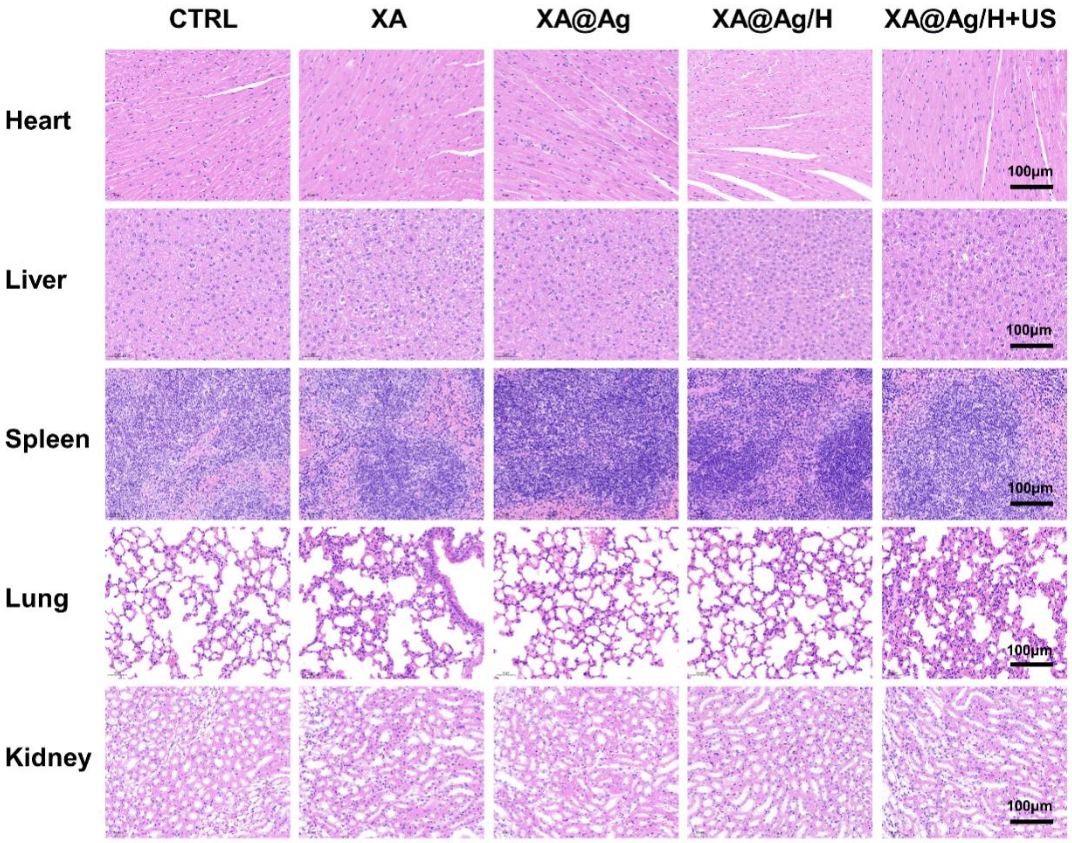

Figure S16. The main organs (heart, liver, spleen, lungs, and kidneys) of mice were subjected to HE staining following the administration of various hydrogels for the treatment of diabetic wounds for 12 days.
